# Utilizing ADMET Analysis and Molecular Docking to Elucidate the Neuroprotective Mechanisms of a Cannabis-Containing Herbal Remedy (Suk-Saiyasna) in Inhibiting Acetylcholinesterase

**DOI:** 10.3390/ijms26073189

**Published:** 2025-03-29

**Authors:** Suwimon Sumontri, Wanna Eiamart, Sarin Tadtong, Weerasak Samee

**Affiliations:** 1 Department of Pharmaceutical Chemistry, Faculty of Pharmacy, Srinakharinwirot University, Nakhon Nayok 26120, Thailand; suwimon.puk@g.swu.ac.th; 2 Technical and Planning Division, Department of Thai Traditional and Alternative Medicine, Ministry of Public Health, Nonthaburi 11000, Thailand; 3 Chula Pharmacokinetic Research Center, Faculty of Medicine, Chulalongkorn University, Bangkok 10330, Thailand; wanna.eiamart@g.swu.ac.th; 4 Department of Pharmacognosy, Faculty of Pharmacy, Srinakharinwirot University, Nakhon Nayok 26120, Thailand; sarin@g.swu.ac.th

**Keywords:** Alzheimer, cannabis, antioxidant, acetylcholinesterase, docking, flavonoids, terpenoids, alkaloids, neuroprotective

## Abstract

Alzheimer’s disease is characterized by the degeneration of cholinergic neurons, which is primarily driven by the acetylcholinesterase (AChE) enzyme and oxidative stress. This study investigated the therapeutic potential of the cannabis-containing herbal remedy Suk-Saiyasna in alleviating amyloid β42 (Aβ42)-induced cytotoxicity in SH-SY5Y cells. The DPPH radical-scavenging activity and inhibitory effects on AChE were evaluated in vitro. The AChE inhibitory potential of 167 ligands, including cannabinoids, flavonoids, terpenoids, and alkaloids derived from Suk-Saiyasna, was assessed using ADMET analysis and molecular docking techniques. The results demonstrated that the Suk-Saiyasna extract exhibited a DPPH radical scavenging effect with an IC_50_ value of 27.40 ± 1.15 µg/mL and notable AChE inhibitory activity with an IC_50_ of 1.25 ± 0.35 mg/mL. Importantly, at a concentration of 1 µg/mL, the extract significantly protected cells from Aβ42-induced stress compared to controls. Docking studies revealed that delta-9-tetrahydrocannabinol (Δ^9^-THC), mesuaferrone B, piperine, β-sitosterol, and chlorogenic acid exhibited substantial binding affinities to AChE, surpassing reference drugs like galantamine and rivastigmine. Furthermore, in silico ADMET predictions indicated that Δ^9^-THC and piperine possessed favorable pharmacokinetic profiles, including solubility, absorption, and blood–brain barrier permeability, with no neurotoxicity or carcinogenicity associated with Δ^9^-THC.

## 1. Introduction

Alzheimer’s disease is a complex neurodegenerative disorder characterized by cognitive decline and memory loss and is primarily attributed to the degeneration of cholinergic neurons and a significant reduction in acetylcholine levels in the brain. One of the principal therapeutic strategies for managing Alzheimer’s disease involves the use of acetylcholinesterase (AChE) inhibitors, which aim to prevent the breakdown of acetylcholine, thereby enhancing cholinergic transmission and alleviating symptoms associated with cognitive impairment [1,2,3]. AChE is a crucial enzyme that hydrolyzes acetylcholine into choline and acetic acid, and its inhibition is considered a cornerstone in the symptomatic treatment of Alzheimer’s disease [4,5]. Several AChE inhibitors have been approved for clinical use, including donepezil, rivastigmine, and galantamine. These agents have demonstrated efficacy in improving cognitive function in patients with mild to moderate Alzheimer’s disease [6,7]. Rivastigmine is noted for its dual inhibition of both AChE and butyrylcholinesterase (BChE), which is beneficial, given that butyrylcholinesterase activity tends to increase in the later stages of Alzheimer’s disease, while AChE activity decreases [8]. This dual action may provide a more comprehensive approach to managing the cholinergic deficits observed in Alzheimer’s disease patients [8,9]. Research has also explored the potential of natural compounds and plant-derived substances as AChE inhibitors. For instance, studies have shown that certain natural peptides and alkaloids exhibit significant AChE inhibitory activity, suggesting that they may serve as alternative therapeutic agents or adjuncts to conventional treatments [10]. Additionally, compounds such as β–carotene have been investigated for their neuroprotective properties and their ability to modulate AChE activity, potentially offering new avenues for Alzheimer’s disease treatment [11]. The cholinergic hypothesis posits that the cognitive decline in Alzheimer’s disease is primarily due to reduced acetylcholine synthesis and increased AChE activity, leading to diminished cholinergic signaling [12,13]. This hypothesis underscores the importance of AChE inhibitors in restoring cholinergic function and improving cognitive outcomes in Alzheimer’s disease patients. Furthermore, ongoing research into novel AChE inhibitors, including synthetic compounds and natural extracts, continues to expand the therapeutic landscape for Alzheimer’s disease, with the aim of not only alleviating symptoms but also addressing the underlying pathophysiological mechanisms of the disease [14,15]. The inhibition of AChE remains a vital strategy in the management of Alzheimer’s disease. By increasing the availability of acetylcholine in the synaptic cleft, AChE inhibitors can enhance cholinergic transmission and provide symptomatic relief. The exploration of both synthetic and natural AChE inhibitors offers promising prospects for improving treatment outcomes in individuals affected by this debilitating condition.

Molecular docking studies have become an essential tool in the field of drug discovery, particularly for identifying potential inhibitors of AChE, an enzyme critically involved in the pathophysiology of Alzheimer’s disease. AChE inhibitors are designed to enhance acetylcholine levels in the brain, thereby improving cholinergic neurotransmission and alleviating cognitive deficits associated with Alzheimer’s disease [16]. The molecular docking approach allows researchers to predict how small molecules, or ligands, interact with AChE at the molecular level, providing insights into binding affinities and interaction patterns that are crucial for the development of effective therapeutics. Recent studies have demonstrated the utility of molecular docking in evaluating various natural and synthetic compounds as potential AChE inhibitors. For instance, El-Nashar et al. conducted docking studies with essential oils from *Acca sellowiana*, revealing interactions with the AChE active site and confirming the binding affinity of these compounds [17]. Similarly, the work by Moustafa et al. highlighted the importance of molecular docking in assessing the interactions of *Cymbopogon citratus* essential oils with AChE, indicating strong binding affinities that suggest potential therapeutic applications [18]. These findings underscore the role of molecular docking as a predictive tool in identifying promising candidates for further experimental validation. Moreover, the molecular docking studies often correlate well with in vitro activity assays, reinforcing the reliability of computational predictions [19,20,21]. Molecular docking serves as a powerful computational method in the search for effective AChE inhibitors, facilitating the identification and optimization of compounds that may lead to new therapeutic options for Alzheimer’s disease. The ability to predict binding interactions and affinities not only accelerates the drug discovery process but also provides a deeper understanding of the molecular mechanisms underlying AChE inhibition [22,23]. Recent advancements in computational methods have significantly improved the accuracy and efficiency of such studies. For example, a recent study in 2025 employed artificial intelligence (AI)-based virtual screening to identify novel AChE inhibitors, illustrating the role of AI in expediting drug discovery processes [24].

ADMET (absorption, distribution, metabolism, excretion, and toxicity) prediction plays a critical role in the development of AChE inhibitors derived from natural products, particularly in therapeutic strategies against Alzheimer’s disease. As AChE inhibitors, these natural compounds are of interest due to their potential to enhance the availability of acetylcholine in the synaptic cleft, potentially improving cognitive function. However, the commercialization of natural product-derived AChE inhibitors is hindered by pharmacokinetic challenges, necessitating thorough ADMET evaluations early in the drug development process. Research indicates that natural products exhibit significant structural diversity, making them excellent candidates for AChE inhibition. Various plant–derived alkaloids have documented AChE inhibitory properties [25]. To complement these biological activities, comprehensive ADMET profiling is essential. This profiling often involves assessing the physicochemical properties of compounds against established drug-likeness criteria, such as Lipinski’s Rule of Five. Evaluations include parameters like molecular weight, lipophilicity (LogP), hydrogen bond donors (HBD), and hydrogen bond acceptors (HBA), which suggest a high likelihood of favorable oral bioavailability that is crucial for therapeutic effectiveness [26].

Suk-Saiyasna is a Thai traditional medicinal formulation known for its sleep-enhancing properties. Officially delisted from the controlled substances category, it was incorporated into the National List of Essential Herbal Medicines in 2021. The mechanism of action is attributed to its ability to disperse wind in the cerebral and abdominal regions, thereby preventing wind stagnation and promoting optimal circulation. Consequently, this aids in reducing sleep onset latency, extending sleep duration, and improving appetite [27]. Recent studies indicate that Suk-Saiyasna may also enhance the effects of drugs targeting the γ–aminobutyric acid type A (GABAA) receptors, thereby influencing sleep and sedation outcomes [28]. The formulation consists of twelve herbal ingredients in specific proportions: camphor (one part), *Azadirachta indica* A. Juss. leaves (two parts), *Kleinhovia hospita* L. root (three parts), *Cinnamomum bejolghota* (Buch.–Ham.) Sweet bark (four parts), *Nigella sativa* L. seeds (five parts), *Aucklandia lappa* (Decne.) Decne. root (six parts), *Myristica fragrans* Houtt. fruit (seven parts), *Mesua ferrea* L. flowers (eight parts), *Piper nigrum* L. fruit (nine parts), *Zingiber officinale* Roscoe rhizome (ten parts), *Piper retrofractum* Vahl. fruit (eleven parts), and *Cannabis sativa* L. leaves (twelve parts) [27]. Furthermore, a study conducted by Werawattanachai et al. revealed that the extracts of Piper nigrum L. fruit and Piper retrofractum Vahl. fruit possess inhibitory effects on AChE, with IC_50_ values of 11.13 and 14.08 µg/mL, respectively [29]. In addition, Suttithumsatid et al. reported that *Cannabis sativa* L. extract exhibits an AChE inhibitory effect with an IC_50_ value of 52.30 µg/mL [30]. These findings underscore the promising avenues for investigating the neuroprotective effects of the Suk-Saiyasna herbal remedy, particularly through its mechanism of action inhibiting AChE.

This study aims to determine the efficacy of cannabinoids, i.e., THC and CBD, against Alzheimer’s disease. We have demonstrated the antioxidant activity, AChE inhibitory activity, and neuroprotective effects of extracts from the Suk-Saiyasna herbal remedy. Furthermore, we identified potential AChE inhibitors through molecular docking studies of the active constituents within the Suk-Saiyasna herbal remedy. These herbs are promising candidates for the discovery of new AChE inhibitors due to their diverse chemical classes, including alkaloids, cannabinoids, flavonoids, lignans, phenolic acids, sesquiterpenes, stilbenes, and terpenoids. Additionally, a virtual ADMET study supports the selection of new candidate AChE inhibitors from the Suk-Saiyasna herbal remedy.

## 2. Results

### 2.1. Total Phenolics, Total Flavonoids, and Antioxidant Activity of Suk-Saiyasna Herbal Remedy Extracts

Extracts of the Suk-Saiyasna herbal remedy were prepared using a sequential solvent extraction method facilitated by microwave-assisted extraction. The fractions of dichloromethane, ethanol, and water were subsequently evaporated to dryness to determine the total phenolic content (TP), total flavonoid content (TF), and 2,2–diphenyl–1–picrylhydrazyl (DPPH) antioxidant activity. The results presented in Table 1 provided a comprehensive evaluation of the TP, TF, and DPPH antioxidant activity of the extracts. It was found that the extracts of the Suk-Saiyasna herbal remedy in the dichloromethane, ethanol, and water fractions had phenolic concentrations of 15.34 ± 0.35 mg GAE/g, 34.24 ± 0.59 mg GAE/g, and 1.04 ± 0.06 mg GAE/g of extract, respectively. The flavonoid contents exhibited a similar trend relative to the total polyphenol values. The mean total flavonoid content in the dichloromethane, ethanol, and water fractions was determined to be 32.26 ± 1.05 mg QE/g, 61.08 ± 1.67 mg QE/g, and 1.86 ± 0.13 mg QE/g of extract, respectively. Antioxidant activity was assessed using the DPPH scavenging assay, with ascorbic acid serving as the standard substance at concentrations ranging from 0.78 to 25 µg/mL. The IC_50_ for ascorbic acid was established at 6.46 ± 0.29 µg/mL. In the evaluation of the dichloromethane fraction, which was tested at concentrations ranging from 12.5 to 800 µg/mL, the IC_50_ was recorded at 153.93 ± 40.87 µg/mL. Additionally, the IC_50_ for the ethanolic fraction was measured at 27.40 ± 1.51 µg/mL within the same concentration range. However, due to a percentage inhibition of less than 50% for the water extract, the IC_50_ value could not be calculated. Notably, the IC_50_ values for the dichloromethane and ethanolic fractions were found to be higher than that of the standard ascorbic acid.

### 2.2. Neurotoxicity and Neuroprotective Activity of Suk-Saiyasna Herbal Remedy Extracts

**Figure 1:**
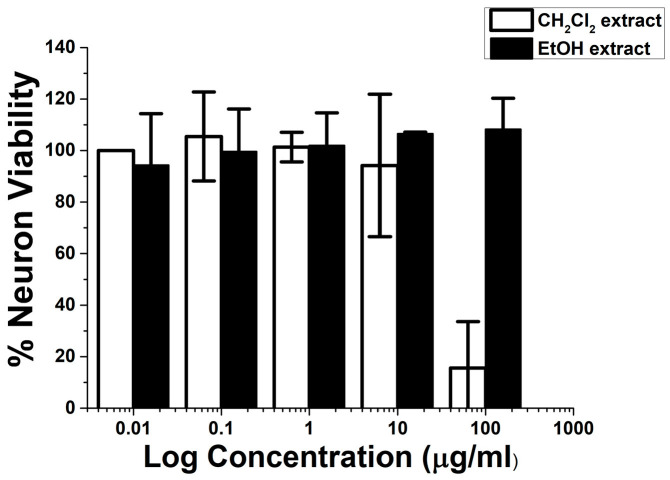
Neurotoxicity of ethanol and dichloromethane extracts of the Suk-Saiyasna herbal remedy on SH–SY5Y cells.

The ethanol and dichloromethane extracts of the Suk-Saiyasna herbal remedy, when evaluated at concentrations ranging from 0.01 to 10 µg/mL, exhibited no signs of neurotoxicity. However, at a concentration of 100 µg/mL, the dichloromethane extract demonstrated neurotoxicity, with neuron viability measured at 15.59 ± 18.05%, while the ethanol extract showed no neurotoxicity and recorded neuron viability at 108.25 ± 12.01% at the same concentration, as illustrated in Figure 1. Notably, at 10 µg/mL, the dichloromethane extract (% neuron viability at 94.19 ± 27.64%) displayed greater neurotoxicity in comparison to the ethanol extract (% neuron viability at 106.56 ± 0.61%), likely due to the wide standard deviation resulting from the poor solubility of the extract in the medium. Consequently, the dichloromethane extract at 1 µg/mL and the ethanol extract at both 1 µg/mL and 10 µg/mL were selected for further investigation of their neuroprotective capabilities against Aβ42-induced neurotoxicity.

**Figure 2:**
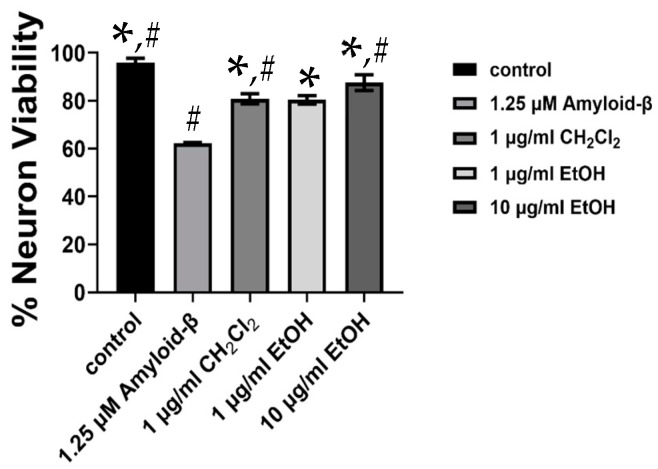
Neuroprotective effects of 1 and 10 µg/mL of ethanol extract and 1 µg/mL of dichloromethane extract on SH–SY5Y cells against 1.25 µM Aβ42. * *p* < 0.05 compared to Aβ42, and # *p* < 0.05 compared to 1 µg/mL ethanol extract.

The ethanol and dichloromethane extracts of the Suk-Saiyasna herbal remedy were further examined for their neuroprotective properties against Aβ42, a neurotoxin associated with Alzheimer’s disease. At a concentration of 1 µg/mL, both extracts significantly protected human neuroblastoma SH–SY5Y cells from neuronal death induced by Aβ42, as illustrated in Figure 2. Based on these findings, it was proposed that the Suk-Saiyasna herbal remedy exerted a protective effect on SH–SY5Y cells against Aβ42-induced neurotoxicity, thereby highlighting its potential neuroprotective properties.

### 2.3. Acetylcholinesterase Inhibitory Activity of Suk-Saiyasna Herbal Renedy Extracts

**Figure 3:**
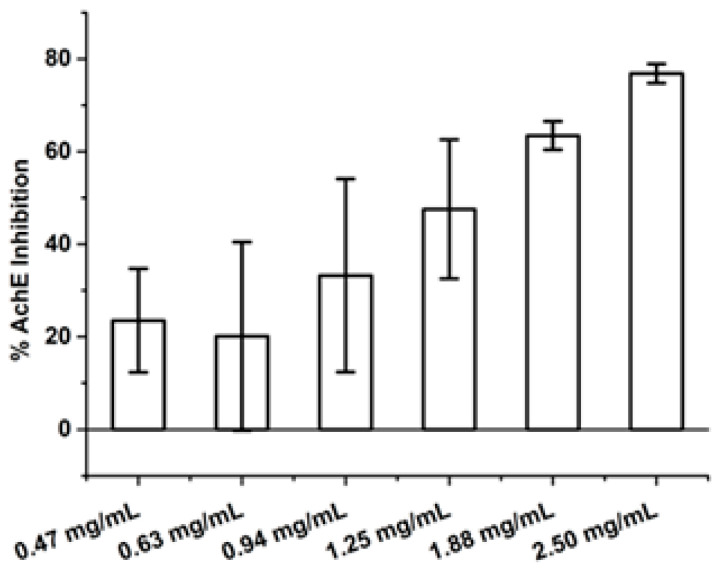
Average acetylcholinesterase inhibitory activity of the Suk-Saiyasna herbal remedy dichloromethane extract at concentrations ranging from 0.47 to 2.5 mg/mL.

The ethanol and dichloromethane extracts of the Suk-Saiyasna herbal remedy at a concentration of 2.5 mg/mL demonstrated AChE inhibitory activities of 59.04 ± 0.35% and 76.84 ± 2.09%, respectively. At a lower concentration of 1.88 mg/mL, the ethanol and dichloromethane extracts exhibited AChE inhibitory activities of 11.74 ± 20.33% and 50.46 ± 22.86%, respectively. Consequently, only the dichloromethane extract was selected for further assays to determine its IC_50_ value for AChE inhibitory activity. The results indicated that the dichloromethane extract of the Suk-Saiyasna herbal remedy exhibited an IC_50_ of 1.25 ± 0.35 mg/mL. Figure 3 illustrates the average acetylcholinesterase inhibitory activity of the dichloromethane extract at concentrations ranging from 0.47 to 2.5 mg/mL. The positive control, galantamine, at a concentration of 1 µM, demonstrated AChE inhibitory activity of 78.33 ± 2.48%.

The results indicated that the Suk-Saiyasna herbal remedy possessed the ability to protect cultured neurons from Aβ42-induced neurotoxicity and could enhance the neurotransmitter acetylcholine by inhibiting the activity of the enzyme AChE. This remedy demonstrated a multi-target approach for addressing key hallmarks of Alzheimer’s disease. Consequently, it may have served as a potential herbal remedy to improve the quality of life for individuals suffering from Alzheimer’s disease.

### 2.4. In-Silico Structural Analysis and Identification of Acethylcholinesterase Binding Site

**Figure 4:**
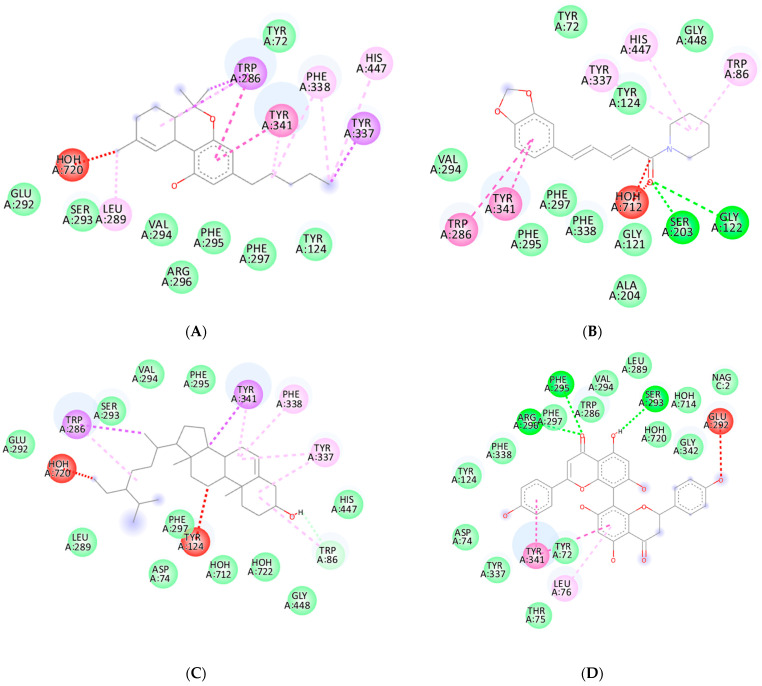

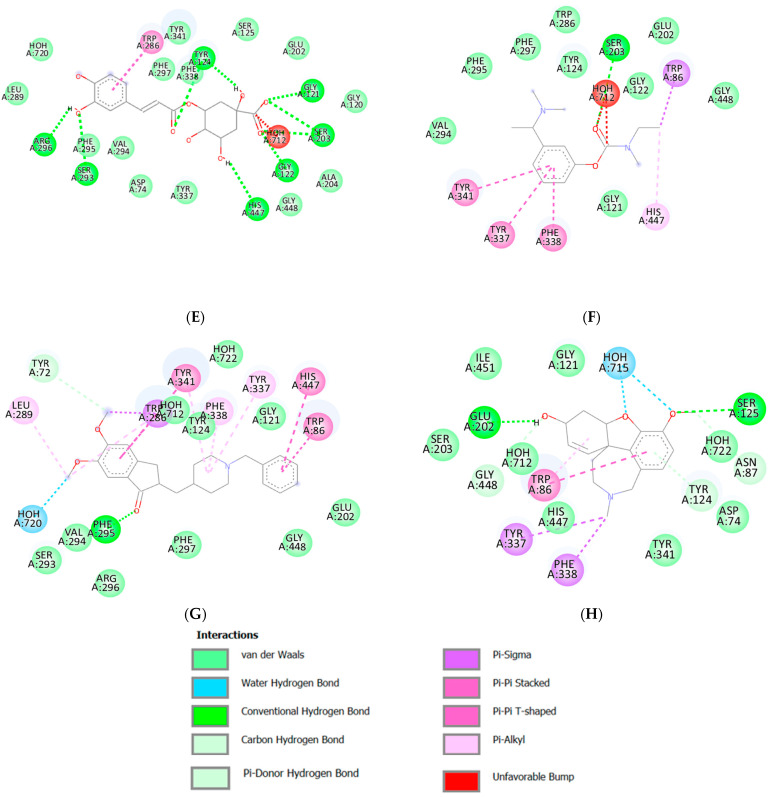
Two-dimensional binding representation of acetylcholinesterase with different ligands: (**A**) Δ^9^-THC, (**B**) piperine, (**C**) β–sitosterol, (**D**) mesuaferrone B, (**E**) chlorogenic acid, (**F**) rivastigmine, (**G**) donepezil, and (**H**) galantamine.

The three-dimensional structural data of acetylcholinesterase (PDB: 7E3H) were employed as the target proteins for docking studies aimed at assessing the binding interactions of the proposed inhibitors. Donepezil was selected as the reference compound due to its established clinical efficacy in the treatment of Alzheimer’s disease. As a reversible AChE inhibitor, donepezil functioned by binding to the active site of the enzyme, effectively preventing the degradation of acetylcholine and enhancing cholinergic neurotransmission [31]. To ensure a comprehensive exploration of potential binding interactions, a grid box was defined to encompass the target protein, centered at coordinates x = −40.919, y = 35.841, and z = −29.382, with dimensions of 60 Å × 60 Å × 60 Å. Grid-based molecular docking techniques were widely employed in drug discovery to accurately predict ligand–receptor interactions, particularly for AChE inhibitors [32]. As illustrated in Figure 4G, the docking analysis revealed that donepezil interacted with AChE through a combination of hydrophobic, pi-stacking, and hydrogen bonding interactions. Specifically, donepezil formed pi–alkyl interactions with residues TYR337 and PHE338, pi–pi stacking interactions with TRP86, TYR341, and HIS447, and van der Waals interactions with residues GLY121, TYR124, GLU202, SER293, VAL294, ARG296, PHE297, and GLY448. Additionally, it established hydrogen bonds with PHE295A, which played a crucial role in stabilizing the ligand–receptor complex. These interactions aligned with previous molecular docking studies that demonstrated donepezil’s capacity to bind within the active gorge of AChE, thereby contributing to its inhibitory activity [33]. The role of pi–pi and pi–alkyl interactions in ligand stabilization was well-documented in molecular docking studies, highlighting their importance in acetylcholinesterase inhibition [34]. Pi-stacking interactions, particularly with TRP86 and TYR341, were essential for stabilizing inhibitors in the active site and were often observed in clinically approved drugs, such as galantamine and rivastigmine [35]. The formation of hydrogen bonds, such as the one observed between donepezil and PHE295A, was also a crucial determinant of binding affinity and had been reported in various computational and crystallographic studies on acetylcholinesterase inhibitors [36]. These findings provided valuable insights into the molecular interactions governing acetylcholinesterase inhibition and underscored the significance of hydrophobic and hydrogen bonding interactions in designing potential inhibitors.

### 2.5. Binding Affinity of Selected Compounds in Suk-Saiyasna Herbal Renedy

The 184 herbal ingredients in the Suk-Saiyasna herbal remedy included *Azadirachta indica* A. Juss. (8 compounds), *Kleinhovia hospita* L. (4 compounds), *Cinnamomum bejolghota* (Buch.–Ham.) Sweet (55 compounds), *Nigella sativa* L. (23 compounds), *Aucklandia lappa* Decne. (5 compounds), *Myristica fragrans* Houtt. (11 compounds), *Mesua ferrea* L. (6 compounds), *Piper nigrum* L. and *Piper retrofractum* Vahl. (17 compounds), *Zingiber officinale* Roscoe. (27 compounds), and *Cannabis sativa* L. (28 compounds). A total of 17 compounds were duplicated, resulting in 167 unique compounds utilized in this study. AutoDock Vina was employed for molecular docking to assess the interactions between the ligands and AChE. The binding affinities of the 167 compounds, along with three reference drugs (donepezil, galantamine, and rivastigmine), are presented in Table 2. The affinity of ligand–target interactions was characterized by binding energy values ranging from −12 to −8 kcal/mol, indicating their potential as acetylcholinesterase inhibitors. Notably, 79 out of the 167 compounds exhibited binding energies less than −8 kcal/mol. According to the study by Ivanova (2022), a binding energy threshold of −8.0 kcal/mol was deemed unsuitable for the reliable identification of potentially active compounds for AChE, suggesting that a much lower binding energy value should be employed [37]. The use of a binding energy threshold lower than −10.0 kcal/mol as a selection criterion could significantly reduce the size of the final set of compounds and increase the number of “lead” compounds, with 18 out of the 167 compounds exhibiting ∆G ≤ −10.0 kcal/mol.

The docking studies revealed that delta–9–tetrahydrocannabinol (∆^9^-THC), mesuaferrone B, piperine, β–sitosterol, and chlorogenic acid displayed significant binding affinities to acetylcholinesterase, with respective values of −10.3, −11.5, −10.5, −10.2, and −10.0 kcal/mol. These compounds, categorized as cannabinoids, flavonoids, alkaloids, terpenoids, and phenolic acids, ranked among the highest of the studied ligands. Importantly, their binding affinities surpassed those of galantamine (−9.6 kcal/mol) and rivastigmine (−8.2 kcal/mol), while remaining comparable to that of donepezil (−11.7 kcal/mol).

### 2.6. Protein–Ligand Interactions of the Top Ligands from Alkaloids, Cannabinoids, Flavonoids, Terpenoids, and Phenolic Phytochemicals

The binding interactions between the ligands and the protein targets were analyzed using Discovery Studio Visualizer. The results, depicted in Figure 4, demonstrated that hydrogen bond interactions were established between SER203 and GLY122 with piperidine; ARG296, PHE295, and SER293 with mesuaferrone B; GLY121, GLY122, TYR124, SER203, SER293, ARG296, and HIS447 with chlorogenic acid; PHE295 with donepezil; SER125 and GLU202 with galantamine; and SER203 with rivastigmine. In contrast, Δ^9^-THC and β–sitosterol did not interact with the acetylcholinesterase binding site through hydrogen bonding. Most ligand–acetylcholinesterase interactions were facilitated through hydrophobic mechanisms, including van der Waals interactions, pi–sigma interactions, pi–stacked interactions, pi–pi T-shaped interactions, and pi–alkyl interactions. The hydrophobic interactions involving the amino acids closely mirrored those of donepezil, forming a triangular arrangement among the amino acids TYR124, VAL295, and PHE338 (or GLY448). A summary of the contacted amino acids for each ligand is presented in Table 3.

### 2.7. ADME Predictions of the Top Ligands from Alkaloids, Cannabinoids, Flavonoids, Terpenoids, and Phenolic Phytochemical Classes

To further investigate the potential of Δ^9^-THC, piperine, β–sitosterol, mesuaferrone B, and chlorogenic acid as orally bioavailable candidates in comparison to commercial acetylcholinesterase inhibitors, their absorption, distribution, metabolism, and excretion (ADME) properties were evaluated using the Swiss ADME webserver [38] and the admetSAR [39] free online tool. As presented in Table 4, Δ^9^-THC, piperine, and β–sitosterol exhibited favorable drug-like properties, fully adhering to Veber’s Rule, while mesuaferrone B and chlorogenic acid violated this rule due to their polar surface area exceeding 140 Å^2^. Similar to the reference drugs, piperine completely complied with Lipinski’s Rule of Five.

Δ^9^-THC showed one violation of Lipinski’s Rule of Five, specifically regarding the LogP value, which should be below 5; it slightly exceeded this threshold with a LogP value of 5.75. β–Sitosterol, like Δ^9^-THC, also presented a LogP value of 8.02, indicating its insolubility in water and inability to penetrate the blood–brain barrier (BBB). Mesuaferrone B exhibited two violations of Lipinski’s Rule of Five, specifically with a molecular weight greater than 500 and a hydrogen bond donor count exceeding five, which prevented its penetration of the BBB. Chlorogenic acid also violated Lipinski’s Rule of Five due to the number of hydrogen bond donors exceeding five, in addition to a LogP value of −0.75, both of which restricted its ability to cross the BBB. These findings suggest that, despite a minor deviation, Δ^9^-THC might still be a promising candidate for further development as an oral therapeutic agent, similar to piperine. However, β–sitosterol, mesuaferrone B, and chlorogenic acid were deemed unsuitable for neuroprotective applications because of their inability to penetrate the BBB.

**Figure 5:**
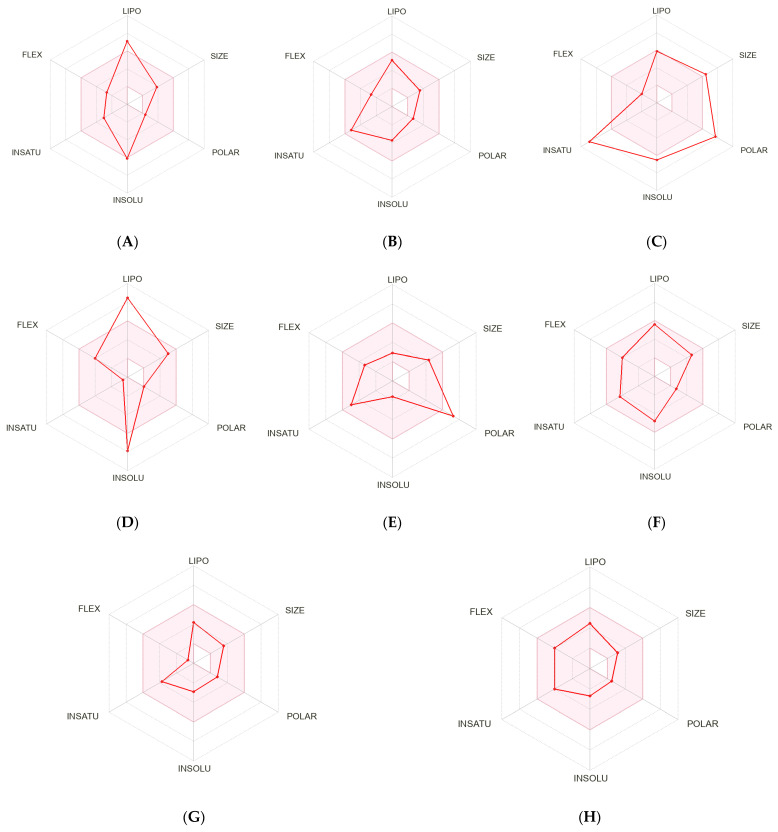
Pink-zone oral bioavailability radars for (**A**) Δ^9^-THC, (**B**) piperine, (**C**) mesuaferrone B, (**D**) β–sitosterol, (**E**) chlorogenic acid, (**F**) donepezil, (**G**) galantamine, and (**H**) rivastigmine.

The bioavailability profiles of eight compounds are depicted in Figure 5. This radar plot consists of six axes representing critical characteristics of oral bioavailability: solubility (INSOLU), flexibility (FLEX), size (SIZE), lipophilicity (LIPO), saturation (IN-SATU), and polarity (POLAR). The pink region in the radar plot denotes the optimal property parameters for oral bioavailability, while the red lines outline the scaffolds of the compounds.

Piperine (Figure 5B) and the reference drugs (Figure 5F–H) are situated within the pink area, indicating that these designed scaffolds possess acceptable estimated oral bioavailability. In contrast, the lipophilicity of Δ^9^-THC (Figure 5A) falls outside the pink area, suggesting reduced water solubility that negatively impacts its oral bioavailability, necessitating advanced formulations to enhance this characteristic. Chlorogenic acid (Figure 5E) exhibits polarity outside the pink area, indicating excessive polarity that could hinder gastrointestinal absorption and, consequently, oral bioavailability.

Mesuaferrone B demonstrated low oral bioavailability due to multiple factors, including molecular size, solubility, saturation, and polarity. Similarly, β–sitosterol exhibited low oral bioavailability attributed to its high lipophilicity and poor water solubility. A critical factor affecting the oral bioavailability of β-sitosterol was its physico-chemical properties, particularly its high lipophilicity. Burayag et al. indicated that β-sitosterol had a log *p* value exceeding nine, which limited its aqueous solubility in the gastrointestinal tract and subsequently decreased absorption efficiency [40]. Adamu et al. also confirmed that β-sitosterol exhibited low intestinal absorption properties among the other phytochemicals evaluated [41]. Clinical studies showed that the actual intestinal absorption rate of β-sitosterol was notably low; for instance, one study reported absorption values of only 0.42% following oral administration compared to 0.63% for campesterol [42]. The low bioavailability of Mesuaferrone B, characterized by its molecular weight of 540.47 Da and six hydrogen bond donors, could be attributed to multiple factors affecting absorption in biological systems. Generally, compounds with higher molecular weights tend to exhibit lower bioavailability due to their inability to easily cross cell membranes. As noted by Wang et al., large molecules often struggle to penetrate biological barriers, leading to diminished absorption characteristics [43]. The presence of numerous hydrogen bonds could enhance intermolecular interactions and reduce solubility, thereby limiting bioavailability [44]. Specifically, compounds with many hydrogen bond donors tend to form strong interactions with water, resulting in challenges in achieving effective partitioning into lipid membranes.

**Figure 6:**
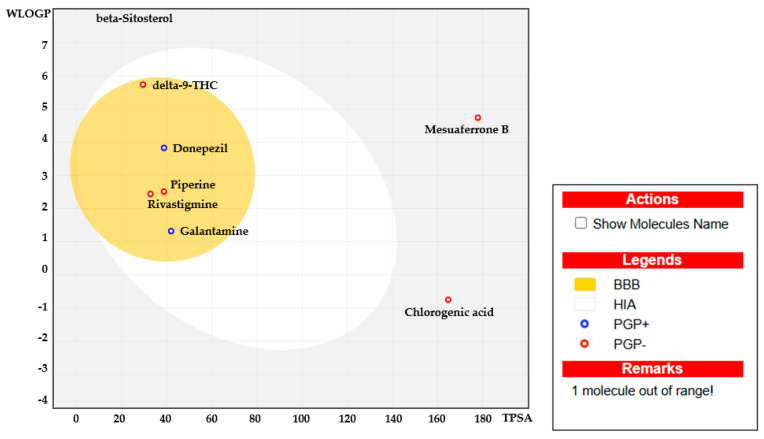
Boiled Egg plot showing the water partition coefficient (WlogP) vs. the topological polar surface area (TPSA) of Δ^9^-THC, piperine, mesuaferrone B, β–sitosterol, chlorogenic acid, donepezil, galantamine, and rivastigmine.

Computational bioavailability properties were assessed through Boiled Egg analysis (WlogP vs. TPSA), where the yellow region (yolk) represented the physicochemical space of molecules with the highest probability of permeating the blood–brain barrier (BBB). As illustrated in Figure 6, both piperine and Δ^9^-THC were able to penetrate the BBB, similar to the reference drugs. In contrast, mesuaferrone B, β–sitosterol, and chlorogenic acid were situated outside the egg area, indicating their inability to penetrate the BBB due to an excessively high LogP value for β–sitosterol and an excessively high polar surface area for mesuaferrone B and chlorogenic acid. Additionally, donepezil and galantamine exhibited active efflux from the central nervous system and gastrointestinal lumen, mediated by P-glycoprotein (PGP+), as indicated by the blue dot. In contrast, the other compounds were classified as non-substrates for P-glycoprotein (PGP−), as denoted by the red dot [45].

### 2.8. In Silico Toxicity Prediction of Selected Compounds

The essential requirement for compounds intended for human use was that they must be non-toxic. Consequently, the toxicity of several compounds—namely Δ^9^-THC, piperine, mesuaferrone B, β–sitosterol, and chlorogenic acid—along with a reference drug, was assessed in silico utilizing Protox-II software. The toxicity assessment in Table 5 classified these substances into six distinct toxicity classes, with Class 1 representing the most lethal and toxic compounds (LD_50_ ≤ 5) and Class 6 indicating non-toxic compounds (LD_50_ > 5000 mg/kg). Piperine was categorized in Class 4, with an LD_50_ value of 330 mg/kg, while Δ^9^-THC also fell into Class 4, exhibiting an LD_50_ of 500 mg/kg. β–Sitosterol was similarly classified in Class 4 with an LD_50_ of 890 mg/kg. Mesuaferrone B and chlorogenic acid were both placed in Class 5, with an LD_50_ of 5000 mg/kg for each. The reference drugs donepezil, galantamine, and rivastigmine were categorized into Classes 4, 3, and 4, respectively. All compounds were determined to be inactive concerning hepatotoxicity. However, piperine, donepezil, and galantamine exhibited active carcinogenicity, while Δ^9^-THC, mesuaferrone B, and chlorogenic acid were inactive regarding neurotoxicity. The theoretical data regarding biological activity and toxicity suggest that Δ^9^-THC, mesuaferrone B, and chlorogenic acid could be considered safe for human use due to their non-toxic nature.

## 3. Discussion

Suk-Saiyasna is a cannabis-containing herbal remedy that has been officially removed from the category of controlled substances and was incorporated into the National List of Essential Herbal Medicines in 2021. Its commercial formulations were utilized to enhance sleep quality and improve appetite [27]. Previous research reported that the Δ^9^-THC content in commercial Suk-Saiyasna herbal remedies ranged from 0.00231% to 0.14218%, which was within the legal limit of 0.2% and confirmed that it was below the concentration level associated with addiction. Our earlier research indicated that commercial Suk-Saiyasna herbal remedies contained Δ^9^-THC levels in ranging from 0.00231% to 0.14218%, demonstrating compliance with the legal limit of 0.2% and confirming that these levels were lower than the addictive concentration threshold [46]. To support the widespread use of the Suk-Saiyasna herbal remedy, we conducted research to identify new indications for this remedy. In this study, the complete formulation of Suk-Saiyasna scavenged DPPH radicals, inhibited AChE, and protected neuronal cells. The molecular docking studies demonstrated that chlorogenic acid, β–sitosterol, piperine, Δ^9^-THC, and mesuaferrone B exhibited high binding affinities with AChE, which corresponded with previous in vitro studies [30,47,48,49,50,51].

Evidence from in vitro studies on the target compounds is as follows: chlorogenic acid exhibited a pronounced ability to inhibit AChE and BChE activities. The study demonstrated a dose-dependent suppression of these enzymes, with IC_50_ values of 8.01 μg/mL for AChE and 6.3 μg/mL for BChE, indicating a statistically significant effect (*p* < 0.05). These results suggest that chlorogenic acid may play a crucial role in modulating enzyme activity, potentially offering neuroprotective benefits and therapeutic applications [47]. β-Sitosterol exhibited potential anti-Alzheimer’s properties by targeting various disease-related aspects, including anti-AChE and antioxidant activity. It inhibited AChE and BChE activities in a concentration-dependent manner, with IC_50_ values of 55 μg/mL and 50 μg/mL, respectively, and demonstrated significant antioxidant properties. Behavioral studies, including Spatial Working Memory (SWM), Y-maze, and Balance Beam tests, showed improvements in cognitive deficits, short-term memory, and locomotor abilities. In vivo studies indicated that β-sitosterol effectively crossed the blood–brain barrier, which contrasted with findings from the in silico study. Additionally, it inhibited cholinesterase-metabolizing enzymes and functioned as a free radical scavenger [48]. Piperine, along with its derivatives (piperic acid and piperic ester), demonstrated significant AChE inhibitory activity both in vitro and through molecular docking simulations. Piperine inhibited AChE activity by 46.5%, while piperic acid and piperic ester exhibited inhibition rates of 50.6% and 63.6%, respectively. These findings suggest their potential as candidates for developing AChE inhibitors targeted at treating Alzheimer’s disease [49]. Direct in vitro evidence for the AChE inhibitory activity of Δ^9^-THC is lacking. However, cannabis extracts with high cannabinoid concentrations may exhibit significant AChE inhibition, with an IC_50_ value of 52.3 ± 8.13 μg/mL (containing 8.1% *w*/*w* CBD and 52.2% *w*/*w* THC). These results suggest that, while THC may function as an adjunct, CBD is the primary cannabinoid responsible for AChE inhibition. Extracts rich in cannabinoids, specifically those containing at least 8% *w*/*w* CBD and low THC content, could be considered as alternative AChE inhibitors [30]. There is no direct evidence that Mesuaferrone B alone exhibits AChE inhibitory activity in vitro. However, ethanol extracts from *Mesua ferrea* Linn. flowers, which contain Mesuaferrone A and B as major constituents, have demonstrated anti-AChE activity relevant to the pathogenesis of Alzheimer’s disease [50,51]. The Suk-Saiyasna herbal remedy demonstrated significant potential for neuroprotection, primarily attributed to its inhibitory activity against AChE and its antioxidant properties. This holistic synergy of cannabinoids and other bioactive compounds presents a promising avenue for addressing cognitive regeneration and quantitative analysis of other compounds to control the quality of the Suk-Saiyasna herbal remedy relating to neurodegenerative diseases.

## 4. Materials and Methods

### 4.1. Plants and Chemical Reagents

The plant parts, such as leaves of *C. sativa*, were obtained from the Bureau of Drug Narcotics, Department of Medical Sciences, Ministry of Public Health, Thailand. Other plant parts were purchased form herbal shops in Thailand, and preparation of the in–house Suk-Saiyasna remedy involved weighing an herbal powder blend composed of 12 herbal ingredients in specific proportions: Camphor—1 part; *Azadirachta indica* A. Juss. leaves—2 parts; *Kleinhovia hospita* L. root—3 parts; *Cinnamomum bejolghota* (Buch.–Ham.) Sweet. bark—4 parts; *Nigella sativa* L. seed—5 parts; *Aucklandia lappa* (Decne.) Decne. root—6 parts; *Myristica fragrans* Houtt. fruit—7 parts; *Mesua ferrea* L. flowers—8 parts; *Piper nigrum* L. fruit—9 parts; *Zingiber officinale* Roscoe rhizome—10 parts; *Piper retrofractum* Vahl. fruit—11 parts; and *Cannabis sativa* L. leaves—12 parts. Analytical-grade dichloromethane and ethanol were procured from Merck (Boston, MA, USA).

### 4.2. Preparation of Crude Extract

Dichloromethane, ethanol, and water were employed sequentially as extracting solvents. The extraction process utilized a liquid–liquid extraction method, in which 30 g of each sample was mixed with dichloromethane at a ratio of 1:10 (*w*/*v*) and subjected to sonication for 30 min. Following extraction, the mixture was filtered using a vacuum pump to separate the solvent from the solid residue. The solid residue was then re-extracted with ethanol and water, respectively. Subsequently, the resulting fractions from each solvent were evaporated to dryness using a rotary evaporator. The dry weights of the extracts were measured, and they were stored in a refrigerator at −20 degrees Celsius for further analysis.

### 4.3. Determination of Total Phenolic Content

The total phenolic content (TP) was determined using the Folin–Ciocalteu method, with modifications tailored to the specific sample matrix. In this procedure, 1 mL of Folin–Ciocalteu reagent was mixed with 300 µg/mL of the sample extract and allowed to react for 5 min. Following this initial reaction, 1 mL of a 75 mg/mL sodium carbonate (Na_2_CO_3_) solution was added, and the final volume of the mixture was adjusted to 10 mL with distilled water. The reaction mixture was then incubated at room temperature for 90 min to facilitate complete color development. Subsequently, absorbance was measured at 765 nm against a blank. A standard curve for gallic acid was constructed using concentrations ranging from 10 to 40 µM, and TP values were expressed as micrograms of gallic acid equivalents (GAE) per milligram of extract. Each sample was analyzed in triplicate to ensure statistical reliability.

### 4.4. Determination of Total Flavonoid Content

Total flavonoid content (TF) was assessed using a modified colorimetric method. In this assay, 1 mL of an 8 mg/mL aluminum chloride (AlCl_3_) solution was added to 300 µg/mL of the sample extract, and the mixture was vortexed to ensure uniformity. Following a 1-h incubation period, absorbance was measured at 415 nm against a blank to determine flavonoid concentration. A calibration curve was established using quercetin standards at concentrations ranging from 15 to 80 µM. TFC values were expressed as micrograms of quercetin equivalents (QE) per milligram of extract. All analyses were conducted in triplicate to ensure accuracy and consistency.

### 4.5. Evaluation of Antioxidant Activity

The antioxidant activity of the extracts was initially evaluated using the DPPH (2,2–diphenyl–1–picrylhydrazyl) radical scavenging assay, adapted from the methodology outlined in previous research. In this assay, 100 µL of the extract solution was combined with 100 µL of an ethanolic DPPH solution (0.6 mM) and vigorously mixed to ensure thorough interaction. The mixture was allowed to react for 30 min in a dark environment at room temperature. Following the incubation period, the absorbance of the resulting solutions was measured at 520 nm using a spectrophotometer, with a control sample consisting of the same DPPH concentration but without the extract.

Radical scavenging activity (RSA) was calculated as a percentage of DPPH discoloration using the following formula:

RSA (%) = [(Acontrol − Asample)/Acontrol] × 100% (1)

where Acontrol represents the absorbance of the DPPH in methanol, and Asample refers to the absorbance of the DPPH solution mixed with the plant extract. The IC_50_ values were determined through linear regression analysis to indicate antioxidant capacity. All samples were analyzed in triplicate to ensure reliability and accuracy.

### 4.6. Evaluation of Neurotoxicity and Neuroprotective Activity

#### 4.6.1. Cell Culture

SH–SY5Y ATCC CRL2266 was cultured in 1:1 *v*/*v* minimum essential medium (MEM, Gibco, Grand Island, NY, USA): nutrient mixture medium (F12, Gibco, USA) supplemented with 10%v/v fetal bovine serum (FBS, Sigma, St. Louis, MO, USA), 1 mM sodium pyruvate (Gibco, USA), and 1% *v/v* antibiotic–antimycotic solution (Gibco, USA) at 37 °C in a 5% CO_2_ humidified atmosphere [52].

#### 4.6.2. Determination of Toxicity of Suk-Saiyasna Herbal Remedy Extracts

SH–SY5Y cells (1 × 10^4^ cells/well) were cultured in 1:1 *v*/*v* MEM:F12 supplemented with 10% *v*/*v* FBS, 1 mM sodium pyruvate, and 1% *v*/*v* antibiotic–antimycotic solution as the completed medium and were treated with 0.01–100 µg/mL of Suk-Saiyasna herbal remedy ethanol and dichloromethane extracts for 24 h. Untreated cells were cultured in a complete medium. Cell viability was determined using the CCK–8 assay. After incubation of CCK–8 for 2 h, the absorbance was measured at 450 nm using a microplate spectrophotometer. A cell treated with 0.5% *v*/*v* DMSO in completed medium was used as the control. Three independent experiments were performed, and each experiment was run in triplicate. The percentage of cell viability was calculated using the following equation:

% cell viability = OD of treated cell × 100/OD of untreated cell

#### 4.6.3. Determination of Neuroprotective Effect of Suk-Saiyasna Herbal Remedy Extracts on Aβ42-Induced Toxicity in SH–SY5Y Cells

The neuroprotective effect of Suk-Saiyasna herbal remedy ethanol and dichloromethane extracts against amyloid β42 (Aβ42)-induced SH–SY5Y cell toxicity was tested. The cells (1 × 10^4^ cells/well) were cultured as described above. After 24 h, the cells were pretreated with 1, 10 µg/mL of Suk-Saiyasna herbal remedy ethanol extract, and 1 µg/mL of Suk-Saiyasna herbal remedy dichloromethane extract for 1 h before being incubated with 1.25 µM Aβ42 for another 24 h. The control cells were cultured in a complete medium containing 0.5% DMSO. Cell viability was determined using the CCK–8 assay described above. Three independent experiments were performed, and each experiment was run in triplicate [52].

#### 4.6.4. Statistical Analysis

The data are presented as the mean ± standard deviation (SD) and were analyzed using OriginPro 9.0 software. One-way analysis of variance (ANOVA), followed by Fisher’s LSD test (*p* < 0.05), was used to determine differences between groups.

### 4.7. Evaluation of Acetylcholinesterase Inhibitory Activity

The assay was performed by Ellman’s method by using eel’s acetylcholinesterase 0.2 U/mL (Sigma, USA) as the target enzyme and 1.5 mM acetylcholine iodine (ATCI, Sigma, USA) as substrate. An amount of 50 mM of TRIS/HCl (Sigma, USA) buffer with a pH of 8.0 was used as solvent for the assay system. Galantamine (Sigma, USA) in an amount of 1 µg/mL was used as the positive control. The extracts were dissolved and diluted with TRIS/HCl buffer and tested at final concentrations of 0.47–2.5 mg/mL. First, 25 µL of the enzyme and 25 µL of the extracts at various concentrations or galantamine were added into a 96-well plate and incubated at 37 °C for 15 min. Then, 25 µL of 1.5 mM ACTI and 125 µL of 3 mM 5,5–dithio–bis–(2–nitrobenzoic acid) (DTNB, Sigma, USA) were added into the well and further incubated at 37 °C for another 15 min. After that, the reaction mixture was measured for its absorbance at 405 nm. Three independent experiments were performed, and each experiment was run in triplicate [53]. The % inhibition was calculated by using the equation below.

[1 − ((AbsSample − Absblank sample)/(Abscontrol − Absblank control))] × 100

### 4.8. Molecular Docking Studies

The chemical structures of the natural compounds were obtained from PubChem, (https://pubchem.ncbi.nlm.nih.gov/, accessed on 8 November 2024). The complete library of 181 molecules was screened to search for acetylcholinesterase inhibitor. The structure of acetylcholinesterase (PDB ID: 7E3H) was downloaded from Protein Data Bank in RCSB (https://www.rcsb.org/, accessed on 8 November 2024) and was prepared using BIOVIA Discovery Studio Visualizer 4.5 (Dassault Systèmes, Waltham, MA, USA); the PDB file of the crystal structure was prepared on the BIOVIA Discovery Studio Visualizer 4.5. All water molecules within the protein structure were removed, and hydrogen atoms were added using the AutoDockTools. The binding interaction between the ligands and acetylcholinesterase was modeled using AutoDock Vina, which considers the protein as a rigid structure. The docking experiment was performed using the Lamarckian genetic algorithm as a search method. All other parameters were run with default settings for each docking. The grid box size (60 Å × 60 Å × 60 Å) was used with a spacing value of 0.375 Å. The grid center for acetylcholinesterase was applied at the coordinate of −40.919, 35.841 and −29.382. To improve its accuracy, the EXHAUSTIVENESS was set to 50 (default = 8). The poses with the best docking score were compared with the scores of E20 (donepezil) from the crystal structures. The resulting AutoDock Vina output files in PDBQT format recorded the poses generated, while the text data listed binding energies for the corresponding poses. 

#### 4.8.1. Determination of Search Space on Acetylcholinesterase

The docking search site, where ligands explore possible binding interactions with AChE, was determined using AutoDock Tools 1.5.6 (Morris et al., 2009) [54]. This search space is also known as AutoGrid. The center for the 3D AutoGrid box was set at (x: −40.919, y: 35.841, z: −29.382), while the number of points in dimension for AutoGrid box was set at (x: 60, y: 60, z: 60).

#### 4.8.2. Preparation of Target Protein

The structure of acetylcholinesterase (PDB ID: 7E3H) was downloaded from Protein Data Bank in RCSB PDB format. Using Discovery Studio 2017 (Dassault Systèmes BIOVIA, SanDiego, CA, USA), the target structure was optimized by removing water molecules and all other ligands in the complex as they may affect the binding conformation and interactions. Donepezil was among the ligands co-crystallized, which is a type of FDA-approved acetylcholinesterase inhibitor drug. One of the identical chains in the homodimer structure was also deleted to minimize docking run time. Hydrogen atoms were then added and converted to PDBQT files.

#### 4.8.3. Ligand Preparation

The structures of ligands of 167 compounds from *Azadirachta indica* A. Juss., *Kleinhovia hospita* L., *Cinnamomum bejolghota* (Buch.–Ham.) Sweet, *Nigella sativa* L., *Aucklandia lappa* Decne., *Myristica fragrans* Houtt., *Mesua ferrea* L., *Piper nigrum* L., *Zingiber officinale* Roscoe, *Piper retrofractum* Vahl, and *Cannabis sativa* L. were downloaded from PubChem in SDF format. Their 3D form was generated using Discovery Studio 2017 software. All 181 ligands were converted to PDB format using Discovery Studio 2017 followed by conversion to PDBQT format using AutoDock Tools 1.5.6. Hydrogen atoms were added to the structures as well. Through root detection and the setting of rotatable bonds, ligands were designated as flexible during the docking process. This is in line with the induced fit theory that generates a diverse set of ligand poses within the search space of receptor binding site.

### 4.9. Assessment of Drug-Likeness and In Silico ADMET Prediction

Drug-likeness and the ADMET profiles were replicated from the previous study [38,39] using admet structure–activity relationship (admetSAR) 2.0 tool/database. It is available online: http://lmmd.ecust.edu.cn/admetsar2/ (accessed on 1 January 2025), and so is an online version of the SwissADME web tool, which is available at: http://www.swissadme.ch (accessed on 14 January 2025). For this analysis, the Simplified Molecular Input Line Entry System (SMILES) formats of all the ligands were obtained from the PubChem database. Additionally, the Protox–II software was used to predict potential adverse drug effects, including hepatotoxicity, cardiotoxicity, and neurotoxicity [55].

## 5. Conclusions

This study highlighted the potential of the Suk-Saiyasna herbal remedy in developing novel neuroprotective compounds for Alzheimer’s disease. The extracts of Suk-Saiyasna demonstrated significant antioxidant and acetylcholinesterase inhibitory activities, indicating their therapeutic applications. Molecular docking studies identified various active constituents with promising binding affinities, reinforcing their potential as acetylcholinesterase inhibitors. Additionally, ADME predictions indicated favorable properties for Δ^9^-THC and piperine, underscoring their ability to cross the blood–brain barrier, which is crucial for neuroprotective effects. The safety evaluation of the extracts revealed moderate toxicity for piperine and Δ^9^-THC, while mesuaferrone B and chlorogenic acid displayed a safer profile. The inactivity of these compounds regarding hepatotoxicity and neurotoxicity further supported their potential use in therapeutic settings. However, concerns regarding carcinogenicity associated with piperine, donepezil, and galantamine necessitate rigorous safety assessments. Overall, the findings from this research provide a foundation for the future exploration of Suk-Saiyasna as a promising source of natural antioxidants and neuroprotective agents.

## Figures and Tables

**Table 1:** Total phenolic content, total flavonoid content, and DPPH antioxidant activity of Suk-Saiyasna herbal remedy extracts (*n* = 3).

Fractions	TP (mg GAE/g)	TF (mg QE/g)	DPPH IC_50_ (µg/mL)
Dichloromethane	15.34 ± 0.35	32.26 ± 1.05	153.93 ± 4.87
Ethanol	34.24 ± 0.59	61.08 ± 1.67	27.40 ± 1.51
Water	1.04 ± 0.06	1.86 ± 0.13	>800
Ascorbic acid	–	–	6.46 ± 0.29

**Table 2:** Ligands, plant sources, phytochemical classes, and binding affinity values of the 167 ligands in remedy and three reference drugs.

Ligands	Plants	Phytochemical Class	Binding Affinity (kcal/mol)
apigenin	*Nigella sativa* L.	Flavonoids	−10.1
cannflavin A	*Cannabis sativa* L.	Flavonoids	−11.2
cannflavin B	*Cannabis sativa* L.	Flavonoids	−11.3
cannflavin C	*Cannabis sativa* L.	Flavonoids	−10.3
catechin	*Nigella sativa* L.	Flavonoids	−9.3
epicatechin	*Azadirachta indica* A. Juss.	Flavonoids	−10
mesuaferrone A	*Mesua ferrea* L.	Flavonoids	−11.4
mesuaferrone B	*Mesua ferrea* L.	Flavonoids	−11.5
mesuanic acid	*Mesua ferrea* L.	Flavonoids	−9.2
quercetin	*Mesua ferrea* L.	Flavonoids	−9.4
brachyamide A	*Piper nigrum* L., *Piper retrofractum* Vahl	Alkaloids	−6.7
brachystamide B	*Piper nigrum* L., *Piper retrofractum* Vahl	Alkaloids	−6.6
dehydropipernonaline	*Piper nigrum* L., *Piper retrofractum* Vahl	Alkaloids	−10.5
ecgonine	*Azadirachta indica* A. Juss.	Alkaloids	−5.4
guineensine	*Piper nigrum* L., *Piper retrofractum* Vahl	Alkaloids	−7.2
hordenine	*Cannabis sativa* L.	Alkaloids	−6.5
neopellitorine B	*Piper nigrum* L., *Piper retrofractum* Vahl	Alkaloids	−8.1
pellitorine	*Piper nigrum* L., *Piper retrofractum* Vahl	Alkaloids	−7.5
piperanine	*Piper nigrum* L., *Piper retrofractum* Vahl	Alkaloids	−9.8
pipercallosine	*Piper nigrum* L., *Piper retrofractum* Vahl	Alkaloids	−9.3
piperchabamide C	*Piper nigrum* L., *Piper retrofractum* Vahl	Alkaloids	−7.1
piperdardine	*Piper nigrum* L., *Piper retrofractum* Vahl	Alkaloids	−10.3
piperine	*Piper nigrum* L., *Piper retrofractum* Vahl	Alkaloids	−10.5
pipernonaline	*Piper nigrum* L., *Piper retrofractum* Vahl	Alkaloids	−9.7
piperolactam C	*Piper nigrum* L., *Piper retrofractum* Vahl	Alkaloids	−9.1
piperolein B	*Piper nigrum* L., *Piper retrofractum* Vahl	Alkaloids	−9
piperundecalidine	*Piper nigrum* L., *Piper retrofractum* Vahl	Alkaloids	−9.8
retrofractamide B	*Piper nigrum* L., *Piper retrofractum* Vahl	Alkaloids	−7.5
cannabichromene (CBC)	*Cannabis sativa* L.	Cannabinoids	−9.6
cannabichromenic acid (CBCA)	*Cannabis sativa* L.	Cannabinoids	−9.7
cannabichromevarin (CBCV)	*Cannabis sativa* L.	Cannabinoids	−9.9
cannabichromevarinic acid (CBCVA)	*Cannabis sativa* L.	Cannabinoids	−9.8
cannabidiol (CBD)	*Cannabis sativa* L.	Cannabinoids	−8.9
cannabidiolic acid (CBDA)	*Cannabis sativa* L.	Cannabinoids	−9.2
cannabidivarin (CBDV)	*Cannabis sativa* L.	Cannabinoids	−8.7
cannabidivarinic acid (CBDVA)	*Cannabis sativa* L.	Cannabinoids	−8.5
cannabigerol (CBG)	*Cannabis sativa* L.	Cannabinoids	−9
cannabigerovarinic acid (CBGVA)	*Cannabis sativa* L.	Cannabinoids	−9.8
delta9––tetrahydrocannabinol (∆^9^-THC)	*Cannabis sativa* L.	Cannabinoids	−10.3
delta–9–Tetrahydrocannabivarinic acid (THCVA)	*Cannabis sativa* L.	Cannabinoids	−9.8
δ–3–carene	*Cinnamomum bejolghota* (Buch.–Ham.) Sweet	Terpenoids	−6.8
δ–cadinol	*Cinnamomum bejolghota* (Buch.–Ham.) Sweet	Terpenoids	−8.7
β–caryophyllene	*Cinnamomum bejolghota* (Buch.–Ham.) Sweet	Terpenoids	−8.5
α–Copaene	*Myristica fragrans* Houtt.	Terpenoids	−8.7
β–elemene	*Cinnamomum bejolghota* (Buch.–Ham.) Sweet	Terpenoids	−8.5
δ–guaiene	*Cinnamomum bejolghota* (Buch.–Ham.) Sweet	Terpenoids	−8.3
β–pinene	*Cinnamomum bejolghota* (Buch.–Ham.) Sweet	Terpenoids	−6.7
β–selinene	*Cinnamomum bejolghota* (Buch.–Ham.) Sweet	Terpenoids	−9.5
γ–terpinene	*Myristica fragrans* Houtt.	Terpenoids	−7
α–terpineol	*Myristica fragrans* Houtt.	Terpenoids	−6.9
γ–terpineol	*Cinnamomum bejolghota* (Buch.–Ham.) Sweet	Terpenoids	−7
α–*trans*–bergamotene	*Cinnamomum bejolghota* (Buch.–Ham.) Sweet	Terpenoids	−8.5
α–zingiberene	*Cinnamomum bejolghota* (Buch.–Ham.) Sweet	Terpenoids	−8.9
α–humulene	*Cinnamomum bejolghota* (Buch.–Ham.) Sweet	Terpenoids	−8.5
α–selinene	*Cinnamomum bejolghota* (Buch.–Ham.) Sweet	Terpenoids	−9.5
α–terpinene	*Zingiber officinale* Roscoe	Terpenoids	−7.1
α–cadinol	*Cinnamomum bejolghota* (Buch.–Ham.) Sweet	Terpenoids	−8.6
α–panasinsene	*Cinnamomum bejolghota* (Buch.–Ham.) Sweet	Terpenoids	−8.6
α–phellandrene	*Cinnamomum bejolghota* (Buch.–Ham.) Sweet	Terpenoids	−6.9
α–pinene	*Cinnamomum bejolghota* (Buch.–Ham.) Sweet, *Nigella sativa* L.	Terpenoids	−6.7
α–terpineol	*Zingiber officinale* Roscoe	Terpenoids	−6.9
α–thujene	*Cinnamomum bejolghota* (Buch.–Ham.) Sweet	Terpenoids	−6.8
1,8–cineole	*Cinnamomum bejolghota* (Buch.–Ham.) Sweet	Terpenoids	−6.9
13–epi–manoyl oxide	*Cinnamomum bejolghota* (Buch.–Ham.) Sweet	Terpenoids	−8.5
4–terpineol	*Zingiber officinale* Roscoe	Terpenoids	−6.8
7–epi–α–selinene	*Cinnamomum bejolghota* (Buch.–Ham.) Sweet	Terpenoids	−9.2
anethole	*Myristica fragrans* Houtt.	Terpenoids	−6.8
borneol	*Zingiber officinale* Roscoe	Terpenoids	−6.4
camphene	*Cinnamomum bejolghota* (Buch.–Ham.) Sweet	Terpenoids	−6.6
camphor	*Cinnamomum bejolghota* (Buch.–Ham.) Sweet	Terpenoids	−6.8
carvacrol	*Nigella sativa* L.	Terpenoids	−7.1
caryophyllene	*Myristica fragrans* Houtt.	Terpenoids	−8.5
cis–piperitol	*Zingiber officinale* Roscoe	Terpenoids	−6.8
dithymoquinone	*Nigella sativa* L.	Terpenoids	−10.2
E–β–ocimene	*Cinnamomum bejolghota* (Buch.–Ham.) Sweet	Terpenoids	−5.4
endo–fenchol	*Cinnamomum bejolghota* (Buch.–Ham.) Sweet	Terpenoids	−5.7
epi–α–cadinol	*Cinnamomum bejolghota* (Buch.–Ham.) Sweet	Terpenoids	−8.8
geraniol	*Cinnamomum bejolghota* (Buch.–Ham.) Sweet	Terpenoids	−6.7
geranyl acetate	*Cinnamomum bejolghota* (Buch.–Ham.) Sweet	Terpenoids	−7.2
germacrene–D	*Cinnamomum bejolghota* (Buch.–Ham.) Sweet	Terpenoids	−8.6
guaiol	*Cinnamomum bejolghota* (Buch.–Ham.) Sweet	Terpenoids	−9.2
isoborneol	*Cinnamomum bejolghota* (Buch.–Ham.) Sweet	Terpenoids	−6.4
isoelemicin	*Myristica fragrans* Houtt.	Terpenoids	−7.3
limonene	*Cinnamomum bejolghota* (Buch.–Ham.) Sweet	Terpenoids	−6.9
linalool	*Cinnamomum bejolghota* (Buch.–Ham.) Sweet	Terpenoids	−6.2
myrcene	*Cinnamomum bejolghota* (Buch.–Ham.) Sweet	Terpenoids	−6.5
nerol	*Zingiber officinale* Roscoe	Terpenoids	−6.2
neryl acetate	*Cinnamomum bejolghota* (Buch.–Ham.) Sweet	Terpenoids	−7.3
p–cymene	*Cinnamomum bejolghota* (Buch.–Ham.) Sweet, *Nigella sativa* L.	Terpenoids	−7.1
phytol	*Cinnamomum bejolghota* (Buch.–Ham.) Sweet	Terpenoids	−7
sabinene	*Cinnamomum bejolghota* (Buch.–Ham.) Sweet	Terpenoids	−6.7
sclareolide	*Cinnamomum bejolghota* (Buch.–Ham.) Sweet	Terpenoids	−9.3
spathulenol	*Cinnamomum bejolghota* (Buch.–Ham.) Sweet	Terpenoids	−8.1
terpinen–4–ol	*Cinnamomum bejolghota* (Buch.–Ham.) Sweet	Terpenoids	−6.8
terpinolene	*Zingiber officinale* Roscoe	Terpenoids	−7.2
tetradecanoic acid	*Myristica fragrans* Houtt.	Terpenoids	−6.4
thymohydroquinone	*Nigella sativa* L.	Terpenoids	−7.2
thymol	*Nigella sativa* L.	Terpenoids	−7.1
thymoquinone	*Nigella sativa* L.	Terpenoids	−7.4
α–amyrin	*Mesua ferrea* L.	Terpenoids	−9.2
α–terpinene	*Zingiber officinale* Roscoe	Terpenoids	−7.1
α–terpineol	*Zingiber officinale* Roscoe	Terpenoids	−6.9
β–sitosterol	*Mesua ferrea* L.	Terpenoids	−10.2
β–amyrin	*Mesua ferrea* L.	Terpenoids	−9.8
3–acetyl–11–keto–β–boswellic acid	*Azadirachta indica* A. Juss.	Terpenoids	−8.6
betulin	*Azadirachta indica* A. Juss.	Terpenoids	−8.5
caryophyllene oxide	*Azadirachta indica* A. Juss.	Terpenoids	−10
lupeol	*Azadirachta indica* A. Juss.	Terpenoids	−9
cineole	*Zingiber officinale* Roscoe	Terpenoids	−6.9
elemol	*Zingiber officinale* Roscoe	Terpenoids	−8.5
*trans*–nerolidol	*Zingiber officinale* Roscoe	Terpenoids	−7.8
β–Eudesmol	*Zingiber officinale* Roscoe	Terpenoids	−9.8
costunolide	*Aucklandia lappa* (Decne.) Decne.	Terpenoids	−9
cynaropicrin	*Aucklandia lappa* (Decne.) Decne.	Terpenoids	−9.5
dehydrocostus lactone	*Aucklandia lappa* (Decne.) Decne.	Terpenoids	−9.5
dihydrocostunolide	*Aucklandia lappa* (Decne.) Decne.	Terpenoids	−9.3
mokko lactone	*Aucklandia lappa* (Decne.) Decne.	Terpenoids	−9.5
Kleinhospitines A	*Kleinhovia hospita* L.	Terpenoids	−4.7
Kleinhospitines B	*Kleinhovia hospita* L.	Terpenoids	−4.7
Kleinhospitines C	*Kleinhovia hospita* L.	Terpenoids	−4.7
Kleinhospitines D	*Kleinhovia hospita* L.	Terpenoids	−4.7
cannabisin D	*Cannabis sativa* L.	Lignans	−9.7
*N–trans*–caffeoyltyramine	*Cannabis sativa* L.	Lignans	−10.1
*N–trans*–coumaroyltyramine	*Cannabis sativa* L.	Lignans	−10.2
*N–trans*–feruloyltyramine	*Cannabis sativa* L.	Lignans	−9.5
cannabispiradienone	*Cannabis sativa* L.	Stilbenoids	−9.2
cannabispiran	*Cannabis sativa* L.	Stilbenoids	−8.9
cannabistilbene I	*Cannabis sativa* L.	Stilbenoids	−10.1
denbinobin	*Cannabis sativa* L.	Stilbenoids	−9.5
dihydroresveratrol	*Cannabis sativa* L.	Stilbenoids	−9.2
chlorogenic acid	*Nigella sativa* L.	Phenolics	−10
ferulic acid	*Nigella sativa* L.	Phenolics	−7.5
gallic acid	*Nigella sativa* L.	Phenolics	−6.5
*p*–coumaric acid	*Nigella sativa* L.	Phenolics	−7.3
vanillic acid	*Nigella sativa* L.	Phenolics	−6.5
α–amyl cinnamyl alcohol	*Cinnamomum bejolghota* (Buch.–Ham.) Sweet	Alcohol group	−7.7
1–Hexanol	*Cinnamomum bejolghota* (Buch.–Ham.) Sweet	Alcohol group	−4.4
3,7–dimethyloct–6–en–1–yn–3–ol	*Zingiber officinale* Roscoe	Alcohol group	−3.9
3,7–dimethylocta–1,6–dien–3–ol	*Zingiber officinale* Roscoe	Alcohol group	−6.3
3–methylhexan–2–ol	*Zingiber officinale* Roscoe	Alcohol group	−5.1
4–isopropylbenzyl alcohol	*Zingiber officinale* Roscoe	Alcohol group	−6.7
allo–aromadendrene	*Zingiber officinale* Roscoe	Fat hydrocarbon	−8.7
α–cedrene	*Zingiber officinale* Roscoe	Fat hydrocarbon	−8.7
β–sesquiphellandrene	*Zingiber officinale* Roscoe	Fat hydrocarbon	−8.8
β–thujene	*Zingiber officinale* Roscoe	Fat hydrocarbon	−6.7
2,6–dimethylhept–5–enal	*Zingiber officinale* Roscoe	Aldoketones	−6
2–heptanone	*Zingiber officinale* Roscoe	Aldoketones	−5
butanal	*Zingiber officinale* Roscoe	Aldoketones	−3.8
germacrone	*Zingiber officinale* Roscoe	Aldoketones	−7.6
endo–bornyl acetate	*Zingiber officinale* Roscoe	Ester group	−7.5
geranyl propionate	*Zingiber officinale* Roscoe	Ester group	−7.4
neryl acetate	*Zingiber officinale* Roscoe	Ester group	−7.3
sec–butyl acetate	*Zingiber officinale* Roscoe	Ester group	−4.8
lauric acid	*Nigella sativa* L.	Fatty acids	−6.4
linoleic acid	*Nigella sativa* L.	Fatty acids	−7.5
linolenic acid	*Nigella sativa* L.	Fatty acids	−7.1
myristic acid	*Nigella sativa* L.	Fatty acids	−5.8
oleic acid	*Nigella sativa* L.	Fatty acids	−6.7
palmitic acid	*Nigella sativa* L.	Fatty acids	−6.4
stearic acid	*Nigella sativa* L.	Fatty acids	−6.8
hexanal	*Cinnamomum bejolghota* (Buch.–Ham.) Sweet	Aldehyde group	−4.4
limonene aldehyde	*Cinnamomum bejolghota* (Buch.–Ham.) Sweet	Aldehyde group	−7.2
tetradecanal	*Cinnamomum bejolghota* (Buch.–Ham.) Sweet	Aldehyde group	−6.2
*Z*–cinnamaldehyde	*Cinnamomum bejolghota* (Buch.–Ham.) Sweet	Aldehyde group	−7.3
eugenol	*Myristica fragrans* Houtt.	phenylpropanoids	−7
methyl eugenol	*Myristica fragrans* Houtt.	phenylpropanoids	−7.1
*trans*–isoeugenol	*Myristica fragrans* Houtt.	phenylpropanoids	−6.9
Donepezil		–	−11.7
Galantamine		–	−9.6
Rivastigmine		–	−8.2

**Table 3:** Summary of the interactions between Δ^9^-THC, piperine, β–sitosterol, mesuaferrone B, chlorogenic acid, rivastigmine, donepezil, and galantamine with the amino acid residues within the active site of acetylcholinesterase.

Ligand Name	Binding Affinity (kcal/mol)	Amino Acid Residues Involved in Interaction	
		Hydrogen Bond	Hydrophobic Interactions
Δ^9^-THC	−10.3	–	TYR72, TYR124, TRP286, GLU292, SER293, VAL294, PHE295, PHE297, TYR337, PHE338, TYR341, HIS447
Piperine	−10.5	SER203, GLY122	TYR72, TRP86, GLY121, TYR124, TRP286, SER293, PHE295, PHE297, TYR337, PHE338, TYR341, HIS447, GLY448
β–Sitosterol	−10.2	–	ASP74, TRP86, TRP286, LEU289, GLU292, SER293, VAL294, PHE295, PHE297, TYR337, PHE338, TYR341, HIS447, GLY448
Mesuaferrone B	−11.5	ARG296, PHE295, SER293	TYR72, ASP74, THR75, TYR124, TRP286, LEU289, VAL294, PHE297, TYR337, PHE338, GLY342
Chlorogenic acid	−10.0	GLY121, GLY122, TYR124, SER203, SER293, ARG296, HIS447	ASP74, SER125, GLY120, GLU202, ALA204, TRP286, LEU289, VAL294, PHE295, PHE297, TYR337, TYR341, GLY448
Donepezil	−11.7	PHE295	TRP86, GLY121, TYR124, GLU202, TRP286, LEU289, GLU292, SER293, VAL294, ARG296, PHE297, TYR337, PHE338, TYR341, HIS447, GLY448
Galantamine	−9.6	SER125, GLU202	ASP74, TRP86, ASN87, GLY121, TYR124, SER203, TYR337, PHE338, TYR341, HIS447, GLY448, ILE451
Rivastigmine	−8.2	SER203	GLY121, GLY122, TYR124, GLU202, TRP286, VAL294, PHE295, PHE297, TYR337, PHE338, TYR341, HIS447, GLY448

**Table 4:** ADME outcomes of Δ^9^-THC, piperine, mesuaferrone B, β–sitosterol, chlorogenic acid, and the reference drug via Swiss ADME webserver and admetSAR.

Compounds	MW (Da)	HBD	HBA	LogP	TPSA	RB	MR	Lipinski’s Rule of Five	Veber’s Rule	HIA (%)	BBB
Δ^9^-THC	314.46	1	2	5.74	29.46	4	97.91	Yes (4/5)	Yes	High 96.4%	Yes
Piperine	285.34	0	3	2.51	38.77	4	85.47	Yes (5/5)	Yes	High 97.1%	Yes
Mesuaferrone B	540.47	6	10	4.74	177.89	3	144.55	No (3/5)	No	Low 75.5%	No
β–Sitosterol	414.71	1	1	8.02	20.23	6	133.23	Yes (4/5)	Yes	High 87.3%	No
Chlorogenic acid	354.31	6	9	–0.75	164.75	5	83.50	Yes (4/5)	No	Low 53.3%	No
Donepezil	379.49	0	4	3.83	38.77	6	115.31	Yes (5/5)	Yes	High 98.5%	Yes
Galantamine	446.90	1	4	1.32	41.93	1	84.05	Yes (5/5)	Yes	High 97.4%	Yes
Rivastigmine	250.34	0	3	2.44	32.78	6	73.12	Yes (5/5)	Yes	High 93.7%	Yes

Note: molecular weight (MW) ≤ 500, hydrogen bond donors (HBD) ≤ 5, hydrogen bond acceptors (HBA) ≤ 10, rotatable bond count (RB) ≤ 10, molar refractivity (MR) between 40 and 130, LogP ≤ 5, topological polar surface area (TPSA) ≤ 140 Å, high intestinal absorption (HIA) >80% and blood–brain barrier (BBB).

**Table 5:** Toxicity assessment results of Δ^9^-THC, piperine, mesuaferrone B, β–sitosterol, chlorogenic Acid, and the reference drug analyzed using Protox–II software.

Compounds	Hepatotoxicity	Carcinogenicity	Neurotoxicity	Acute Oral Toxicity (LD_50_: mg/kg)	Toxicity Class
Piperine	Inactive	Active	Active	330	4
Δ^9^-THC	Inactive	Inactive	Inactive	500	4
β–Sitosterol	Inactive	Inactive	Active	890	4
Mesuaferrone B	Inactive	Inactive	Inactive	5000	5
Chlorogenic acid	Inactive	Inactive	Inactive	5000	5
Donepezil	Inactive	Active	Active	505	4
Galantamine	Inactive	Active	Active	85	3
Rivastigmine	Inactive	Inactive	Active	1000	4

Note: Toxicity is in the classification form 1–6. Class 1 is the most toxic and lethal and class 6 is non–toxic.

## Data Availability

Data are contained within the article and Supplementary Materials.

## Supplementary Materials

The supporting information can be downloaded at: https://www.mdpi.com/article/10.3390/ijms26073189/s1.

## Abbreviations

The following abbreviations are used in this manuscript:

AChE	Acetylcholinesterase
ACTI	Acetylcholine iodine
ADMET	Absorption, Distribution, Metabolism, Excretion, and Toxicity
Aβ42	Amyloid Beta 42
AlCl_3_	Aluminum chloride
ANOVA	One-way analysis of variance
ARG	Arginine
ASP	Aspartic acid
BBB	Blood–brain barrier
BChE	Butyrylcholinesterase
CCK–8	Cholecystokinin octapeptide
CO_2_	Carbon dioxide
°C	Celsius degree
Da	Dalton
DMSO	Dimethyl sulfoxide
DPPH	2,2–Diphenyl–1–picrylhydrazyl
DTNB	5,5–dithio–bis–(2–nitrobenzoic acid)
FBS	Fetal Bovine Serum
FLEX	Flexibility
g	Gram
GABAA	γ–aminobutyric acid type A
GAE	Gallic acid equivalent
GLU	Glutamine
GLY	Glycine
HIA	High intestinal absorption
HBA	Hydrogen bond acceptors
HBD	Hydrogen bond donors
HCl	Hydrochloric acid
HIS	Histidine
IC_50_	Half–maximal inhibitory concentration
IN-SATU	Saturation
INSOLU	Solubility
kcal	Kilocalorie
kg	Kilogram
LEU	Leucine
LD_50_	Median lethal dose
LIPO	Lipophilicity
LogP	Logarithm of partition coefficient
MEM:F12	Minimum Essential Medium Eagle/F12 complete medium
mL	Milliliter
mol	Mole
MW	Molecular weight
Na_2_CO_3_	Sodium carbonate
OD	Optical density
PDB	Protein data bank
MR	Molar refractivity
PGP	P–glycoprotein
PHE	Phenylalanine
POLAR	Polarity
QE	Quercetin equivalent
RB	Rotatable bond
SD	Standard deviation
RSA	Radical scavenging activity
SER	Serine
Δ^9^-THC	delta–9–Tetrahydrocannabinol
TF	Total flavonoid content
TP	Total phenolic content
TPSA	Topological polar surface area
TRIS	tris(hydroxymethyl)aminomethane
TYR	Tyrosine
VAL	Valine
µg	Micro gram
µM	Micro molar
