# Supplementary material for: Utilizing ADMET Analysis and Molecular Docking to Elucidate the Neuroprotective Mechanisms of a Cannabis-Containing Herbal Remedy (Suk-Saiyasna) in Inhibiting Acetylcholinesterase
Source: Int J Mol Sci. 2025 Mar 29;26(7):3189. doi: 10.3390/ijms26073189 (PMC11989231; doi:10.3390/ijms26073189)
Supplement: Supplementary file 1 [file ijms-26-03189-s001.zip › ijms-3545272-supplementary.pdf]

S1. Smiles structure of 170 studied ligands.

Molecule	PubChem ID	SMILES
apigenin	5280443	C1=CC(=CC=C1C2=CC(=O)C3=C(C=C(C=C3O2)O)O)O
cannflavin A	10071695	CC(=CCC/C(=C/CC1=C(C2=C(C=C1O)OC(=CC2=O)C3=CC(=C(C=C3)O)OC)O)/C)C
cannflavin B	403815	CC(=CCC1=C(C2=C(C=C1O)OC(=CC2=O)C3=CC(=C(C=C3)O)OC)O)C
cannflavin C	25141335	CC(=CCC/C(=C/CC1=C2C(=C(C=C1O)O)C(=O)C=C(O2)C3=CC(=C(C=C3)O)OC)/C)C
catechin	9064	C1[C@@H]([C@H](OC2=CC(=CC(=C21)O)O)C3=CC(=C(C=C3)O)O)O
epicatechin	72276	C1[C@H]([C@H](OC2=CC(=CC(=C21)O)O)C3=CC(=C(C=C3)O)O)O
mesuaferrone A	101324837	C1[C@H](OC2=C(C(=CC(=C2C1=O)O)O)C3=C4C(=C(C=C3O)O)C(=O)C[C@H](O4)C5=CC=C(C=C5)O)C6=CC=C(C=C6)O
mesuaferrone B	90472563	C1[C@H](OC2=C(C(=CC(=C2C1=O)O)O)C3=C(C=C(C4=C3OC(=CC4=O)C5=CC=C(C=C5)O)O)O)C6=CC=C(C=C6)O
mesuanic acid	5319381	C[C@H]1[C@H](OC2C(C1=O)C(=O)C(=C(C2(CC=C(C)C)CC(=C(C)C)CCC(C)C)O)C(CC(=O)O)C3=CC=CC=C3)C
quercetin	5280343	C1=CC(=C(C=C1C2=C(C(=O)C3=C(C=C(C=C3O2)O)O)O)O)O
brachyamide A	14162525	C1CCN(C1)C(=O)/C=C/C=C/CCCCCC/C=C/C2=CC3=C(C=C2)OCO3
brachystamide B	10047263	CC(C)CNC(=O)/C=C/C=C/CCCCCCCC/C=C/C1=CC2=C(C=C1)OCO2
dehydropipernonaline	6439947	C1CCN(CC1)C(=O)/C=C/C=C/CC/C=C/C2=CC3=C(C=C2)OCO3
ecgonine	91460	CN1[C@H]2CC[C@@H]1[C@H]([C@H](C2)O)C(=O)O
guineensine	6442405	CC(C)CNC(=O)/C=C/C=C/CCCCCC/C=C/C1=CC2=C(C=C1)OCO2
hordenine	68313	CN(C)CCC1=CC=C(C=C1)O
neopellitorine B	11118018	CCCCC/C=C/C=C/C(=O)N1CCCCC1
pellitorine	5318516	CCCCC/C=C/C=C/C(=O)NCC(C)C
piperanine	5320618	C1CCN(CC1)C(=O)/C=C/CCC2=CC3=C(C=C2)OCO3
pipercallosine	5372201	CC(C)CNC(=O)/C=C/C=C/CCCCC1=CC2=C(C=C1)OCO2
piperchabamide C	44454018	C1CCN(CC1)C(=O)/C=C/C=C/CCCCCC/C=C/C2=CC3=C(C=C2)OCO3
piperdardine	10086948	C1CCN(CC1)C(=O)/C=C/C=C/CCC2=CC3=C(C=C2)OCO3
piperine	638024	C1CCN(CC1)C(=O)/C=C/C=C/C2=CC3=C(C=C2)OCO3
pipernonaline	9974595	C1CCN(CC1)C(=O)/C=C/CCCC/C=C/C2=CC3=C(C=C2)OCO3
piperolactam C	10881419	COC1=C(C(=C2C3=C1C4=CC=CC=C4C=C3NC2=O)OC)OC

Molecule	PubChem ID	SMILES
piperolein B	21580213	C1CCN(CC1)C(=O)CCCCCC/C=C/C2=CC3=C(C=C2)OCO3
piperundecalidine	44453654	C1CCN(CC1)C(=O)/C=C/C=C/CCCC/C=C/C2=CC3=C(C=C2)OCO3
retrofractamide B	5372162	CC(C)CNC(=O)/C=C/C=C/CCCC/C=C/C1=CC2=C(C=C1)OCO2
cannabichromene (CBC)	30219	CCCCCC1=CC(=C2C=CC(OC2=C1)(C)CCC=C(C)C)O
cannabichromenic acid (CBCA)	3084339	CCCCCC1=CC2=C(C=CC(O2)(C)CCC=C(C)C)C(=C1C(=O)O)O
cannabichromevarin (CBCV)	6451726	CCCC1=CC(=C2C=CC(OC2=C1)(C)CCC=C(C)C)O
cannabichromevarinic acid (CBCVA)	11110322	CCCC1=CC2=C(C=CC(O2)(C)CCC=C(C)C)C(=C1C(=O)O)O
cannabidiol (CBD)	644019	CCCCCC1=CC(=C(C(=C1)O)[C@@H]2C=C(CC[C@H]2C(=C)C)C)O
cannabidiolic acid (CBDA)	160570	CCCCCC1=CC(=C(C(=C1C(=O)O)O)[C@@H]2C=C(CC[C@H]2C(=C)C)C)O
cannabidivarin (CBDV)	11601669	CCCC1=CC(=C(C(=C1)O)[C@@H]2C=C(CC[C@H]2C(=C)C)C)O
cannabidivarinic acid (CBDVA)	59444387	CCCC1=CC(=C(C(=C1C(=O)O)O)[C@@H]2C=C(CC[C@H]2C(=C)C)C)O
cannabigerol (CBG)	5315659	CCCCCC1=CC(=C(C(=C1)O)C/C=C(\C)/CCC=C(C)C)O
cannabigerovarinic acid (CBGVA)	59444383	CCCC1=CC(=C(C(=C1C(=O)O)O)C/C=C(\C)/CCC=C(C)C)O
delta-9-tetrahydrocannabinol (THC)	16078	CCCCCC1=CC(=C2[C@@H]3C=C(CC[C@H]3C(OC2=C1)(C)C)C)O
delta-9 Tetrahydrocannabivarinic acid (THCVA)	59444416	CCCC1=CC2=C([C@@H]3C=C(CC[C@H]3C(O2)(C)C)C)C(=C1C(=O)O)O
δ-3-carene	26049	CC1=CCC2C(C1)C2(C)C
δ-cadinol	3084311	CC1=C[C@H]2[C@@H](CC[C@@]([C@H]2CC1)(C)O)C(C)C
β-caryophyllene	5281515	C/C/1=C\CCC(=C)[C@H]2CC([C@@H]2CC1)(C)C
α-Copaene	19725	CC1=CCC2C3C1C2(CCC3C(C)C)C
β-elemene	6918391	CC(=C)[C@@H]1CC[C@@]([C@@H](C1)C(=C)C)(C)C=C
δ-guaiene	94275	C[C@H]1CCC2=C(CC[C@H](C[C@@H]12)C(=C)C)C
β-pinene	14896	CC1(C2CCC(=C)C1C2)C
β-selinene	442393	CC(=C)[C@@H]1CC[C@]2(CCCC(=C)[C@@H]2C1)C
γ-terpinene	7461	CC1=CCC(=CC1)C(C)C
α-terpineol	17100	CC1=CCC(CC1)C(C)(C)O
γ-terpineol	11467	CC(=C1CCC(CC1)(C)O)C
α-*trans*-bergamotene	86608	CC1=CCC2CC1C2(C)CCC=C(C)C
α-zingiberene	11127403	CC1=CC[C@H](C=C1)[C@H](C)CCC=C(C)C

Molecule	PubChem ID	SMILES
α- humulene	5281520	C/C/1=C\CC(/C=C/C/C(=C/CC1)/C)(C)C
α- selinene	10856614	CC1=CCC[C@]2([C@H]1C[C@@H](CC2)C(=C)C)C
α-terpinene	7462	CC1=CC=C(CC1)C(C)C
α-cadinol	10398656	CC1=C[C@H]2[C@@H](CC[C@@]([C@@H]2CC1)(C)O)C(C)C
α-panasinsene	578929	CC1=CCCC2(C13CC(C3CC2)(C)C)C
α-phellandrene	7460	CC1=CCC(C=C1)C(C)C
α-pinene	6654	CC1=CCC2CC1C2(C)C
α-terpineol	17100	CC1=CCC(CC1)C(C)(C)O
α-thujene	17868	CC1=CCC2(C1C2)C(C)C
1,-8cineole	2758	CC1(C2CCC(O1)(CC2)C)C
13-epi-manoyl oxide	6432025	C[C@@]1(CC[C@@H]2[C@]3(CCCC([C@@H]3CC[C@]2(O1)C)(C)C)C)C=C
4-terpineol	11230	CC1=CCC(CC1)(C(C)C)O
7-epi-α-selinene	10726905	CC1=CCC[C@]2([C@H]1C[C@H](CC2)C(=C)C)C
anethole	637563	C/C=C/C1=CC=C(C=C1)OC
borneol	64685	CC1(C2CCC1(C(C2)O)C)C
camphene	6616	CC1(C2CCC(C2)C1=C)C
camphor	2537	CC1(C2CCC1(C(=O)C2)C)C
carvacrol	10364	CC1=C(C=C(C=C1)C(C)C)O
caryophyllene	5281515	C/C/1=C\CCC(=C)[C@H]2CC([C@@H]2CC1)(C)C
cis-piperitol	85567	CC1=C[C@@H]([C@@H](CC1)C(C)C)O
dithymoquinone	398941	CC(C)C1=CC(=O)C2(C(C1=O)C3(C2C(=O)C(=CC3=O)C(C)C)C)C
E-β-ocimene	5281553	CC(=CC/C=C(\C)/C=C)C
endo-fenchol	6997371	C[C@@]12CC[C@@H](C1)C([C@@H]2O)(C)C
epi-α-cadinol	160799	CC1=C[C@H]2[C@@H](CC[C@]([C@@H]2CC1)(C)O)C(C)C
geraniol	637566	CC(=CCC/C(=C/CO)/C)C
geranyl acetate	1549026	CC(=CCC/C(=C/COC(=O)C)/C)C
germacrene-D	91723653	C/C/1=C/CCC(=C)/C=C\[C@@H](CC1)C(C)C
guaiol	227829	C[C@H]1CC[C@H](CC2=C1CC[C@@H]2C)C(C)(C)O
isoborneol	6321405	C[C@@]12CC[C@@H](C1(C)C)C[C@H]2O
isoelemicin	5318557	C/C=C/C1=CC(=C(C(=C1)OC)OC)OC
limonene	22311	CC1=CCC(CC1)C(=C)C
linalool	6549	CC(=CCCC(C)(C=C)O)C
myrcene	31253	CC(=CCCC(=C)C=C)C
nerol	643820	CC(=CCC/C(=C\CO)/C)C
neryl acetate	1549025	CC(=CCC/C(=C\COC(=O)C)/C)C
p-cymene	7463	CC1=CC=C(C=C1)C(C)C
phytol	5280435	C[C@@H](CCC[C@@H](C)CCC/C(=C/CO)/C)CCCC(C)C
sabinene	18818	CC(C)C12CCC(=C)C1C2
sclareolide	929262	C[C@]12CCCC([C@@H]1CC[C@@]3([C@@H]2CC(=O)O3)C)(C)C
spathulenol	92231	C[C@@]1(CC[C@@H]2[C@@H]1[C@H]3[C@H](C3(C)C)CCC2=C)O
terpinen-4-ol	11230	CC1=CCC(CC1)(C(C)C)O
terpinolene	11463	CC1=CCC(=C(C)C)CC1
tetradecanoic acid	11005	CCCCCCCCCCCCCC(=O)O

Molecule	PubChem ID	SMILES
thymohydroquinone	95779	CC1=CC(=C(C=C1O)C(C)C)O
thymol	6989	CC1=CC(=C(C=C1)C(C)C)O
thymoquinone	10281	CC1=CC(=O)C(=CC1=O)C(C)C
α-amyrin	73170	C[C@@H]1CC[C@@]2(CC[C@@]3(C(=CC[C@H]4[C@]3(CC[C@@H]5[C@@]4(CC[C@@H](C5(C)C)O)C)C)[C@@H]2[C@H]1C)C)C
α-terpinene	7462	CC1=CC=C(CC1)C(C)C
α-terpineol	17100	CC1=CCC(CC1)C(C)(C)O
β-sitosterol	222284	CC[C@H](CC[C@@H](C)[C@H]1CC[C@@H]2[C@@]1(CC[C@H]3[C@H]2CC=C4[C@@]3(CC[C@@H](C4)O)C)C)C(C)C
β-amyrin	73145	C[C@@]12CC[C@@]3(C(=CC[C@H]4[C@]3(CC[C@@H]5[C@@]4(CC[C@@H](C5(C)C)O)C)C)[C@@H]1CC(CC2)(C)C)C
cannabisin D	71448965	COC1=C(C=CC(=C1)[C@@H]2[C@H](C(=CC3=CC(=C(C=C23)O)OC)C(=O)NCCC4=CC=C(C=C4)O)C(=O)NCCC5=CC=C(C=C5)O)O
N-trans-caffeoyltyramine	9994897	C1=CC(=CC=C1CCNC(=O)/C=C/C2=CC(=C(C=C2)O)O)O
N-trans-coumaroyltyramine	5372945	C1=CC(=CC=C1CCNC(=O)/C=C/C2=CC=C(C=C2)O)O
N-trans-feruloyltyramine	5280537	COC1=C(C=CC(=C1)/C=C/C(=O)NCCC2=CC=C(C=C2)O)O
cannabispiradienone	90475437	COC1=CC2=C(C(=C1)O)C3(CC2)C=CC(=O)C=C3
cannabispiran	162936	COC1=CC2=C(C(=C1)O)C3(CCC(=O)CC3)CC2
cannabistilbene I	146349	CC(=CCC1=C(C=CC(=C1)CCC2=CC(=CC(=C2)OC)O)O)C
denbinobin	10423984	COC1=CC(=C2C(=C1)C=CC3=C2C(=O)C(=CC3=O)OC)O
dihydroresveratrol	185914	C1=CC(=CC=C1CCC2=CC(=CC(=C2)O)O)O
chlorogenic acid	1794427	C1[C@H]([C@H]([C@@H](C[C@@]1(C(=O)O)O)OC(=O)/C=C/C2=CC(=C(C=C2)O)O)O)O
ferulic acid	445858	COC1=C(C=CC(=C1)/C=C/C(=O)O)O
gallic acid	370	C1=C(C=C(C(=C1O)O)O)C(=O)O
p-coumaric acid	637542	C1=CC(=CC=C1/C=C/C(=O)O)O
vanillic acid	8468	COC1=C(C=CC(=C1)C(=O)O)O
3-acetyl-11keto-β-boswellic acid	11168203	C[C@@H]1CC[C@@]2(CC[C@@]3(C(=CC(=O)[C@H]4[C@]3(CC[C@@H]5[C@@]4(CC[C@H]([C@]5(C)C(=O)O)OC(=O)C)C)C)[C@@H]2[C@H]1C)C)C
betulin	72326	CC(=C)[C@@H]1CC[C@]2([C@H]1[C@H]3CC[C@@H]4[C@]5(CC[C@@H](C([C@@H]5CC[C@]4([C@@]3(CC2)C)C)(C)C)O)C)CO
caryophyllene oxide	1742210	C[C@@]12CC[C@@H]3[C@H](CC3(C)C)C(=C)CC[C@H]1O2

Molecule	PubChem ID	SMILES
lupeol	259846	CC(=C)[C@@H]1CC[C@]2([C@H]1[C@H]3CC[C@@H]4[C@]5(CC[C@@H](C([C@@H]5CC[C@]4([C@@]3(CC2)C)C)(C)C)O)C)C
α-amyl cinnamyl alcohol	7584	CCCCCC(=CC1=CC=CC=C1)CO
1-Hexanol	8103	CCCCCCO
3,-7dimethyloct--6en--1yn--3ol	62842	CC(=CCCC(C)(C#C)O)C
3,-7dimethylocta-1,-6dien--3ol	6549	CC(=CCCC(C)(C=C)O)C
3-methylhexan-2-ol	16835	CCCC(C)C(C)O
4-isopropylbenzyl alcohol	325	CC(C)C1=CC=C(C=C1)CO
cineole	2758	CC1(C2CCC(O1)(CC2)C)C
elemol	92138	CC(=C)[C@@H]1C[C@@H](CC[C@@]1(C)C=C)C(C)(C)O
trans-nerolidol	5284507	CC(=CCC/C(=C/CCC(C)(C=C)O)/C)C
β-Eudesmol	91457	C[C@]12CCCC(=C)[C@@H]1C[C@@H](CC2)C(C)(C)O
costunolide	5281437	C/C/1=C\CC/C(=C/[C@@H]2[C@@H](CC1)C(=C)C(=O)O2)/C
cynaropicrin	119093	C=C1C[C@@H]([C@@H]2[C@@H]([C@@H]3[C@H]1C[C@@H](C3=C)O)OC(=O)C2=C)OC(=O)C(=C)CO
dehydrocostus lactone	73174	C=C1CC[C@@H]2[C@@H]([C@@H]3[C@H]1CCC3=C)OC(=O)C2=C
dihydrocostunolide	5367639	CC1C2CC/C(=C\CC/C(=C\C2OC1=O)/C)/C
mokko lactone	167495	C[C@H]1[C@@H]2CCC(=C)[C@@H]3CCC(=C)[C@@H]3[C@H]2OC1=O
allo-aromadendrene	42608158	C[C@@H]1CC[C@H]2[C@@H]1C3C(C3(C)C)CCC2=C
α-cedrene	6431015	C[C@@H]1CC[C@@H]2[C@]13CC=C([C@H](C3)C2(C)C)C
β-sesquiphellandrene	12315492	C[C@@H](CCC=C(C)C)[C@H]1CCC(=C)C=C1
β-thujene	520384	CC1C=CC2(C1C2)C(C)C
2,-6dimethylhept--5enal	61016	CC(CCC=C(C)C)C=O
2-heptanone	8051	CCCCCC(=O)C
butanal	261	CCCC=O
germacrone	6436348	C/C/1=C\CC(=C(C)C)C(=O)C/C(=C/CC1)/C
endo-bornyl acetate	93009	CC(=O)O[C@@H]1C[C@@H]2CC[C@]1(C2(C)C)C
geranyl propionate	5355853	CCC(=O)OC/C=C(\C)/CCC=C(C)C
neryl acetate	1549025	CC(=CCC/C(=C\COC(=O)C)/C)C
sec-butyl acetate	7758	CCC(C)OC(=O)C
lauric acid	3893	CCCCCCCCCCCC(=O)O
linoleic acid	5280450	CCCCC/C=C\C/C=C\CCCCCCCC(=O)O

Molecule	PubChem ID	SMILES
linolenic acid	5280934	CC/C=C\C/C=C\C/C=C\CCCCCCCC(=O)O
myristic acid	11005	CCCCCCCCCCCCCC(=O)O
oleic acid	445639	CCCCCCCC/C=C\CCCCCCCC(=O)O
palmitic acid	985	CCCCCCCCCCCCCCCC(=O)O
stearic acid	5281	CCCCCCCCCCCCCCCCCC(=O)O
hexanal	6184	CCCCCC=O
limonene aldehyde	110923	CC1=CCC(CC1)C(C)CC=O
tetradecanal	31291	CCCCCCCCCCCCCC=O
Z-cinnamaldehyde	6428995	C1=CC=C(C=C1)/C=C\C=O
eugenol	3314	COC1=C(C=CC(=C1)CC=C)O
methyl eugenol	7127	COC1=C(C=C(C=C1)CC=C)OC
trans-isoeugenol	853433	C/C=C/C1=CC(=C(C=C1)O)OC
Kleinhospitine A		CC1=C[C@@]2(C=C(C(=O)N2)[C@H]3CC[C@@]4([C@@]3(CC[C@]56[C@H]4CC[C@@H]7[C@]5(C6)C=CC(=O)C7(C)C)C)C)OC1=O
Kleinhospitine B		CC1=C[C@]2(C=C(C(=O)N2)[C@H]3CC[C@@]4([C@@]3(CC[C@]56[C@H]4CC[C@@H]7[C@]5(C6)C=CC(=O)C7(C)C)C)C)OC1=O
Kleinhospitine C		CC1=C[C@@]2(C=C(C(=O)N2)[C@H]3CC[C@@]4([C@@]3(CC[C@@]56[C@H]4CC[C@@H]7[C@@]5(C6)C=CC(=O)C7(C)C)C)C)OC1=O
Kleinhospitine D		CC1=C[C@]2(C=C(C(=O)N2)[C@H]3CC[C@@]4([C@@]3(CC[C@@]56[C@H]4CC[C@@H]7[C@@]5(C6)C=CC(=O)C7(C)C)C)C)OC1=O
Donepezil	3152	COC1=C(C=C2C(=C1)CC(C2=O)CC3CCN(CC3)CC4=CC=CC=C4)OC
Galantamine	9651	CN1CC[C@@]23C=C[C@@H](C[C@@H]2OC4=C(C=CC(=C34)C1)OC)O
Rivastigmine	77991	CCN(C)C(=O)OC1=CC=CC(=C1)[C@H](C)N(C)C
